# Carbonaceous supports for Schiff base metal complexes: Strategies for enhanced catalytic efficiency

**DOI:** 10.1016/j.isci.2026.114688

**Published:** 2026-01-14

**Authors:** Abhishek Maurya

**Affiliations:** 1 Department of Chemistry and Sustainability, School of Basic Sciences, Galgotias University, Greater Noida 203201, India

**Keywords:** Chemical reaction, Catalysis, Organic chemistry, Polymer chemistry

## Abstract

Recent developments in anchoring metal-Schiff base complexes onto carbonaceous polymeric matrices highlight the growing emphasis on using carbonaceous polymers as versatile support materials. The development of supported metal catalysts has gained significant attention due to their potential to combine the advantages of homogeneous and heterogeneous catalysis. This review explores recent advances in the immobilization of metal-Schiff base complexes onto carbonaceous polymeric supports and its application toward organic transformation reactions. This discussion emphasizes diverse strategies for synthesizing composite materials, particularly the incorporation of metal catalysts into solid supports, such as activated carbon, carbon nanotubes, graphene, and Merrifield resin, which has led to enhanced catalytic performance, stability, and reusability. Additionally, the review addresses challenges related to metal leaching and immobilization techniques, offering insights into the potential of composite carbonaceous polymer matrices for the development of more sustainable and industrially applicable catalytic systems.

## Graphical abstract

**Figure:**
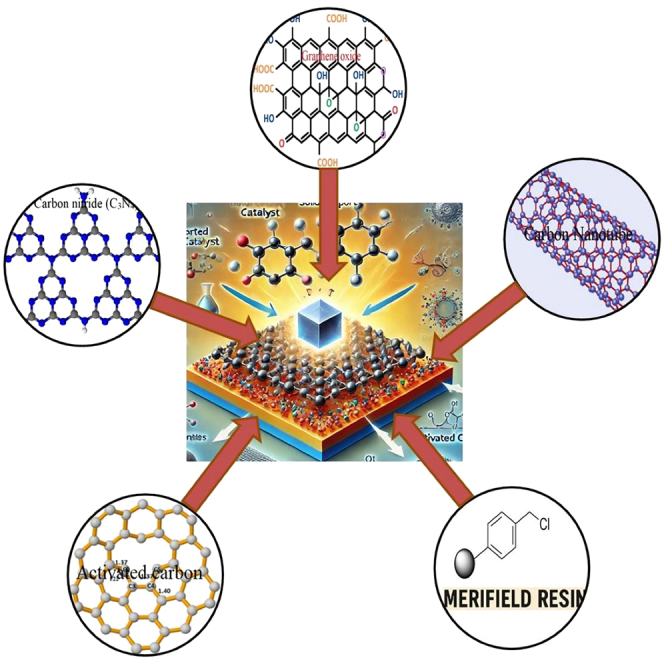

## Introduction

In recent years, considerable research has been devoted to the development of insoluble functionalized polymers with chelating properties, owing to their operational flexibility, practical advantages, and ability to form stable metal-polymer complexes with strong bond energies. A critical factor influencing the stability of these metal complexes is the number of chelate rings; hence, incorporating multidentate ligands into the polymer matrix is of particular interest. While homogeneous catalytic systems offer high activity and selectivity, they suffer from key limitations: (1) difficulty in controlling the selectivity of the desired product, (2) reliance on solution-phase reactions, and (3) challenges in separating and recovering both products and catalysts after the reaction.^1^^,^^2^ These drawbacks have motivated efforts to immobilize homogeneous catalysts such as Schiff base metal complexes onto solid polymeric supports. This strategy aims to bridge the gap between homogeneous and heterogeneous catalysis by combining the efficiency of the former with the practical advantages of the latter. As a result, recent studies have focused on synthesizing supported metal catalysts using a variety of solid and inert materials as supports.^3^^,^^4^^,^^5^^,^^6^^,^^7^^,^^8^ Compared to their solution-phase counterparts, these supported systems offer numerous benefits, including easier separation and recovery, enhanced reusability, and reduced environmental waste.^9^ Consequently, the design and optimization of novel supported metal catalysts have emerged as a central focus in the pursuit of more efficient and sustainable catalytic processes. Table 1 shows the advantages and disadvantages of homogeneous versus heterogeneous catalysts.

**Table 1:** Advantages and disadvantages of homogeneous versus heterogeneous catalysts

SN	Feature	Homogeneous catalysts	Heterogeneous catalysts
1	Catalytic efficiency	high activity and selectivity	generally robust and stable
2	Reaction control	easy to modify for selectivity	easier separation from products
3	Mechanistic study	easier to study at molecular level	harder to study at molecular level
4	Uniform active sites	yes	no
5	Recovery and catalyst reuse	difficult and costly to separate from mixture and not reusable	may suffer from diffusion limitation, less active surface area
6	Stability	less stable at high temperatures or in presence of impurities	more stable at high temperatures but have limited control over active sites
7	Environmental impact	solvents and ligands may be toxic or require special disposal	less environmental impact

When early transition metal complexes are molecularly anchored onto catalyst supports, they generate well-defined surface-bound species that act as highly active and selective heterogeneous catalysts for a wide range of chemical transformations. This capability has driven sustained interest in developing new, efficient, and environmentally friendly transition metal catalysts, a key objective in modern catalysis research.^10^^,^^11^^,^^12^^,^^13^ A particularly promising direction involves the use of earth-abundant, cost-effective metal complexes, which offer practical advantages for large-scale industrial applications.^14^^,^^15^^,^^16^ Central to the performance of these metal complexes is the design of their ligand systems, which critically influence their structure and reactivity. Among the various ligand classes, Schiff base ligands stand out due to their ease of synthesis, structural diversity, and ability to form stable and versatile metal complexes.^17^^,^^18^^,^^19^^,^^20^ These complexes have found widespread applications across numerous catalytic processes, including polymerization,^21^^,^^22^^,^^23^ oxidation of organic substrates,^24^^,^^25^ ketone reduction,^26^ aldol reactions,^27^ Henry reactions,^28^^,^^29^ alkene epoxidation,^30^^,^^31^^,^^32^ Diels-Alder reactions,^33^ alkene metathesis,^34^ and alkene hydrosilylation.^35^ Beyond catalysis, Schiff base complexes also exhibit significant biological activity, such as antibacterial, antifungal, anticancer, and anti-inflammatory properties.^36^^,^^37^^,^^38^^,^^39^^,^^40^^,^^41^ As a result, Schiff base-supported transition metal catalysts have become increasingly important in both academic research and industrial applications, particularly within the polymer and fine chemical industries.

The concept of supported homogeneous catalysis, which seeks to integrate the advantages of both homogeneous and heterogeneous systems, has emerged as a promising approach in the field of catalysis. By combining the high activity and selectivity characteristic of homogeneous catalysts with the ease of separation and reusability typical of heterogeneous systems, supported catalysts offer practical benefits for a variety of applications. A wide range of strategies, both covalent and non-covalent, have been explored to immobilize metal complexes onto diverse support materials. In particular, transition metal Schiff base complexes have attracted increasing attention for immobilization on supports, such as carbon-based polymers,^42^ silica matrices,^43^ mesoporous materials,^44^ and mixed oxides like SiO_2_ and Al_2_O_3_.^45^^,^^46^ Among these strategies, covalent bonding remains the most widely adopted method for anchoring metal complexes to solid supports.^47^^,^^48^ However, despite its potential, the covalent immobilization of homogeneous catalysts onto carbonaceous polymeric supports has been relatively underexplored.^49^^,^^50^^,^^51^^,^^52^^,^^53^^,^^54^ In this context, the present study introduces a simple and effective method for transforming a homogeneous Schiff base metal catalyst into a heterogeneous system via covalent attachment to an carbonaceous solid support, while also evaluating its recyclability and performance.^55^^,^^56^^,^^57^^,^^58^^,^^59^ Although supported catalysts are theoretically expected to outperform their homogeneous counterparts in terms of practicality, especially in large-scale applications their industrial use remains limited.^60^^,^^61^ One contributing factor is the widespread use of commercially available polymeric supports that are not specifically engineered for catalyst immobilization, often resulting in suboptimal catalytic behavior. Furthermore, it is commonly, though perhaps mistakenly, assumed that the performance of a homogeneous catalyst will remain unchanged after immobilization. In reality, the polymeric matrix can influence the activity and selectivity of the catalyst, sometimes detrimentally leading to unexpected or reduced efficiency compared to solution-phase behavior.

In this review, we aim to highlight recent noteworthy examples that demonstrate how the support matrix can enhance the overall efficiency of immobilized catalysts. While some older references are included for their relevance to specific aspects, this work does not attempt to comprehensively cover the literature in this field. Instead, it presents key examples that illustrate the main concepts discussed. The current challenge is no longer just immobilizing catalysts for easier recovery and reuse but developing advanced materials and methodologies that enhance catalytic properties, potentially surpassing those of their soluble counterparts. These effects will be analyzed with a focus on three key factors: activity, stability, and selectivity. For clarity and to provide a well-defined basis for comparison, this review will specifically examine the use of carbonaceous polymeric matrices as catalyst supports. The use of silica and other non-carbonaceous materials as supports falls outside the scope of this work, though other reviews discussing the impact of non-carbonaceous supports on immobilized catalyst efficiency are available in the literature.^62^^,^^63^^,^^64^^,^^65^ Although examples of such systems remain limited, efforts to utilize carbon material matrices to enhance catalytic efficiency, have shown significant success. While catalysts supported on carbon polymer matrices have seen rapid development in recent years, they are not explored in detail in this review, as they fall outside its primary scope. However, carbon materials have garnered increasing attention due to their high surface area and structural diversity, making them excellent candidates for use as catalyst supports.^66^

## Concept of heterogeneous catalysis

Chris Adams, a contributor to the North American Catalysis Society, states that catalysis plays a crucial role in the global economy, with approximately 35% of the world’s GDP relying on it.^67^ Additionally, over 90% of industrial chemicals are produced through catalytic processes.^68^^,^^69^ This highlights the essential role of catalysts in industrial and economic development. With the vast number of catalysts documented in the literature, they can be categorized based on their composition, applications, structure, and state of aggregation.^70^ Among various classification methods, Sabatier was the first to divide catalysts into two main types: homogeneous and heterogeneous.^71^ The primary distinction between homogeneous and heterogeneous catalysis lies in the physical state of the catalyst relative to the reactants in a chemical reaction. A vast array of homogeneous catalysts has been documented in the literature for their industrial and synthetic significance. These include catalysts used in hydroformylation (rhodium- and cobalt-based complexes),^72^ hydrogenation (rhodium-based Wilkinson’s catalyst),^73^^,^^74^ the water-gas shift reaction (iron, rhodium, or ruthenium-based catalysts),^75^ the Monsanto process (rhodium-based catalyst),^76^ the Cativa process (iridium-containing catalyst),^77^^,^^78^ olefin polymerization (Ziegler-Natta catalysts),^79^ olefin metathesis (Grubbs catalyst),^80^ the Wacker process (palladium(II) and copper(II) complexes),^81^ the Halcon process (molybdenum-based catalyst),^82^ and Sharpless dihydroxylation (osmium-based catalysts),^83^^,^^84^ among others. Numerous homogeneous catalytic systems featuring metal complexes have been reported, where the metal center is surrounded by an organic framework to enhance stability and catalytic performance.^85^^,^^86^^,^^87^^,^^88^^,^^89^^,^^90^^,^^91^^,^^92^^,^^93^^,^^94^^,^^95^^,^^96^^,^^97^^,^^98^^,^^99^^,^^100^

The homogeneous nature of these catalysts allows for easy characterization using various physicochemical and spectroscopic techniques, which aid in identifying reactive species and predicting reaction mechanisms. Despite their advantages, homogeneous catalysts pose challenges in recovery and recyclability, which can impact their economic feasibility in industrial processes. To address these limitations, heterogeneous catalysts have been introduced as an alternative solution. Thus, in heterogeneous catalysis, only specific sites on the catalyst’s surface, known as active sites, are responsible for facilitating the catalytic reaction. Therefore, heterogeneous catalysts are designed and synthesized to maximize surface area per unit of catalyst, as catalytic reactions primarily occur at the surface. Modern heterogeneous catalysis employs various solid matrices, such as metal gauzes, metal films, or metal complexes, to anchor metals due to their high surface area, cost-effectiveness, and improved catalytic activity. An ideal solid support for catalysts should be inexpensive, possess a high melting point, and remain chemically and catalytically inert. Currently, the most widely used catalytic supports include zeolites, alumina, silica, carbon nanotubes, graphene, activated carbon, polystyrene beads, and polymers.^45^^,^^99^^,^^100^ The selection of an appropriate solid support is crucial in heterogeneous catalysis, as it facilitates the effective interaction of reactants and products by enabling easy adsorption and desorption. Typically, heterogeneous catalytic reactions involve a sequence of steps, including molecular adsorption onto the catalyst surface, the reaction between the adsorbed reactants, and the subsequent desorption of the products. In 1796, Von Marum introduced the first heterogeneous catalyst for the dehydrogenation of alcohols. Heterogeneous catalysts play a vital role in industrial applications due to their efficiency, selectivity, and ability to facilitate large-scale production, particularly in the energy and chemical industries.^101^ Numerous large-scale industrial processes rely on heterogeneous catalysis, as documented in the literature. Examples include the contact process, catalyzed by vanadium oxides for sulfuric acid synthesis; the Haber-Bosch process, which uses iron oxides on alumina for ammonia production; hydrogen production via steam reforming catalyzed by nickel or K_2_O; olefin polymerization through Ziegler-Natta catalysts; and petroleum desulfurization using molybdenum-cobalt (Mo-Co) catalysts on an alumina surface.^99^^,^^100^^,^^102^^,^^103^ The key advantages of heterogeneous catalysts include high stability, ease of separation, cost-effectiveness, and reusability, making them indispensable for industrial processes.

## Development of hybrid catalytic systems (heterogenization of homogeneous system)

Beyond the advantages and limitations of both homogeneous and heterogeneous catalysis, researchers are focusing on hybrid catalytic systems that integrate the benefits of heterogeneous catalysts into homogeneous systems while minimizing their drawbacks. One effective approach to achieving this is the heterogenization of homogeneous catalysts, which enhances thermal stability, improves activity and selectivity, and allows for easier recovery and recyclability. The concept of immobilizing homogeneous catalysts onto solid supports was first introduced in the 19th century.^104^ Two primary methods for heterogenization have been reported: (1) anchoring ligands onto a solid support followed by metalation, and (2) directly attaching metal complexes onto the solid support. The selection of a suitable solid support is crucial, as it must exhibit high thermal stability and be chemically and catalytically inert. Solid supports used in heterogenization are generally classified into two categories: carbonaceous and non-carbonaceous. Advantages and disadvantages of carbonaceous and non-carbonaceous solid supports is shown in Table 2.

**Table 2:** Advantages and disadvantages of carbonaceous and non-carbonaceous solid supports

Support Type	Advantages	Disadvantages
Carbonaceous supports	• high surface area and porosity • rich in functional groups • good electrical conductivity • strong metal-support interaction • excellent interaction with organic solvents • tunable surface and easy surface functionalization	• costly synthesis • tendency to aggregate or bundle • limited thermal stability • hydrophobic nature
Non-carbonaceous supports	• high thermal and chemical stability • abundant and inexpensive • well-defined porous structures	• lower electrical conductivity • brittle or fragile • diffusion limitations in microporous materials • leach under harsh conditions

**Figure 1:**
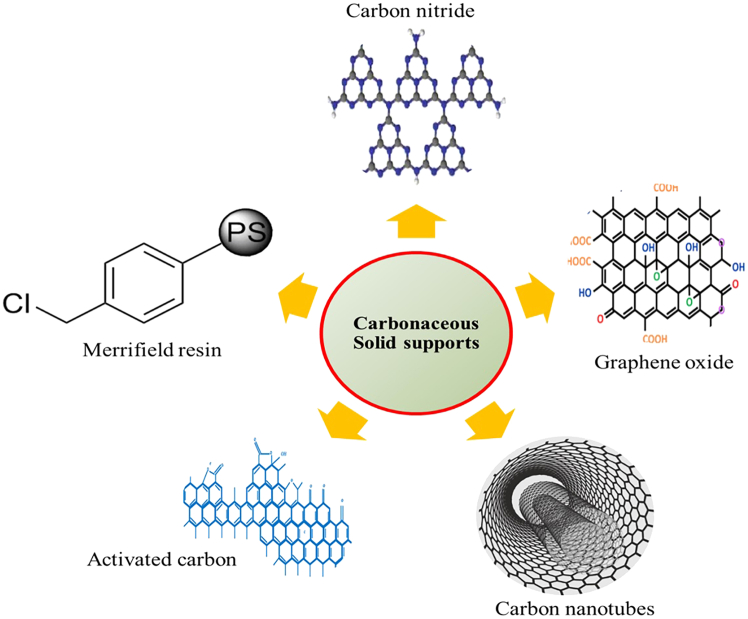
Different types of carbonaceous solid supports

In this review, we prioritize carbonaceous solid supports due to their high surface area, porosity, stability, tunable hydrophobicity and polarity, ease of functionalization, efficient isolation and recovery, strong chemical inertness, excellent interaction with organic solvents, and wide availability. In addition to their numerous advantages, carbonaceous solid supports possess diverse functional groups containing heteroatoms, such as oxygen (O), sulfur (S), nitrogen (N), and halogens (X), along with a higher surface area compared to non-carbonaceous supports like silica and alumina.^105^ Due to their porous structure and chemical properties, carbonaceous solid supports are well-suited for the heterogenization of homogeneous catalysts using chemisorption techniques.^106^^,^^107^ Furthermore, since most industrial synthesis and large-scale chemical transformations occur in organic solvents, these supports interact effectively with the solvents, facilitating an efficient reaction pathway between reactants and catalysts. Recently, several types of carbonaceous solid supports have been developed and synthesized for heterogeneous catalysis. These include graphitic carbon nitride,^108^^,^^109^ activated carbon,^110^ graphene oxide,^111^ carbon nanotubes,^112^^,^^113^ and Merrifield resin.^3^^,^^114^^,^^115^ The Figure 1 shown various carbonaceous solid support.^120^^,^^123^^,^^124^^,^^125^^,^^126^ However, these carbonaceous solid support also have their advantages and disadvantages, which shown in Table 3. Based on the various beneficial properties of carbonaceous materials, researchers are increasingly focusing on their applications nowadays.

**Table 3:** Advantages and disadvantages of various carbonaceous solid supports

Material	Advantages	Disadvantages
Graphene oxide (GO)	• high surface area • excellent dispersibility • abundant functional groups • facilitates strong metal/support interaction	• tendency to aggregate • cost-intensive synthesis • limited thermal stability in air
Carbon nanotubes (CNTs)	• high electrical conductivity • large surface area • good mechanical strength • facilitates electron transfer	• difficult to functionalize uniformly • expensive • prone to bundling
Carbon nitride (g-C_3_N_4_)	• tunable structure • high thermal and chemical stability • nitrogen-rich surface allows better coordination	• poor conductivity • moderate surface area
Activated carbon (AC)	• cost-effective • high porosity • large surface area • widely available	• lacks uniform functional groups • poor mechanical strength • impurities possible
Merrifield resin	• easy functionalization • high loading capacity • reusable	• swelling in organic solvents • limited thermal stability • non-conductive

Currently, numerous organic transformation reactions in industries are carried out using carbonaceous solid-supported heterogeneous catalysts. These include hydrogen transfer, C-H activation, desulfurization,^116^^,^^117^ hydrogenation,^118^ the Mannich reaction, fuel cell reactions,^119^ oxidation,^120^^,^^121^ and oxidative amination,^122^ among others.

## Activated Carbon as a solid support in heterogeneous catalysis

Activated carbon, also known as activated charcoal, is a non-crystalline, highly porous carbon-based material widely used in adsorption and chemical reactions due to its microporous structure and large surface area.^127^ Adsorption studies indicate that a single gram of activated carbon can have a surface area exceeding 3000 m^2^. Its applications span various industries, including air and water purification, sewage treatment, metal extraction, solvent recovery, medicine, compressed air filtration, methane and hydrogen storage, and even teeth whitening.^128^^,^^129^ Due to its environmentally friendly nature and extensive surface area, activated carbon serves as an excellent solid support in heterogeneous catalysis.^130^ Additionally, the surface of activated carbon can be easily functionalized, making it reactive toward different reagents, which facilitates the immobilization of metal complexes. Numerous studies in the literature discuss various methods for anchoring metal complexes onto activated carbon, highlighting its significance in catalytic applications.

**Scheme 1:**
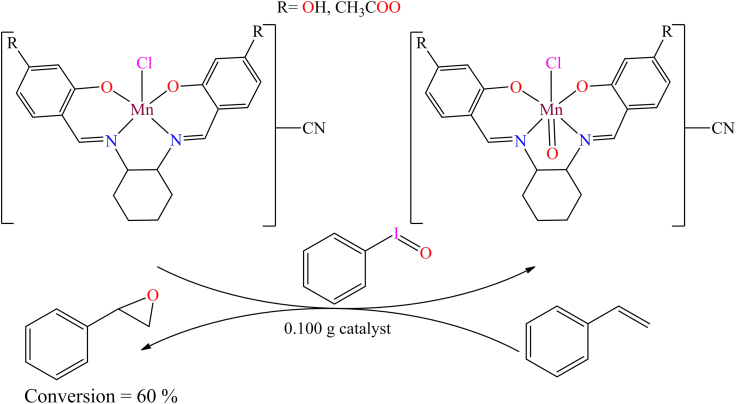
Manganese(III) Schiff base complex immobilized on commercial activated carbon with hydroxyl groups (0.500 mmol of styrene, 0.500 mmol of chlorobenzene (GC internal standard), 0.100 g of catalyst, and 0.250 mmol of iodosylbenzene in 5.00 cm^3^ of acetonitrile) Redrawn based on ref.^131^

Although, Ana Rosa Silva et al. reported the immobilization of a manganese(III) Schiff base complex onto commercial activated carbon functionalized with hydroxyl groups.^131^ In the presence of iodosylbenzene, the manganese(III) Schiff base complex readily oxidizes into an oxo-manganese species (Scheme 1). The oxo-manganese complex showed catalytic activity in the epoxidation of styrene, achieving over 68% conversion and 90% selectivity under optimized conditions: 0.500 mmol styrene, 0.500 mmol chlorobenzene (GC internal standard), 0.100 g catalyst, and 0.250 mmol iodosylbenzene in 5.00 cm^3^ of acetonitrile. The study further revealed that all heterogeneous catalysts displayed activity and chemoselectivity comparable to their homogeneous counterparts, except for the complex supported on nitric acid-oxidized activated carbon, which showed the lowest performance due to the catalytic influence of the support.

**Scheme 2:**
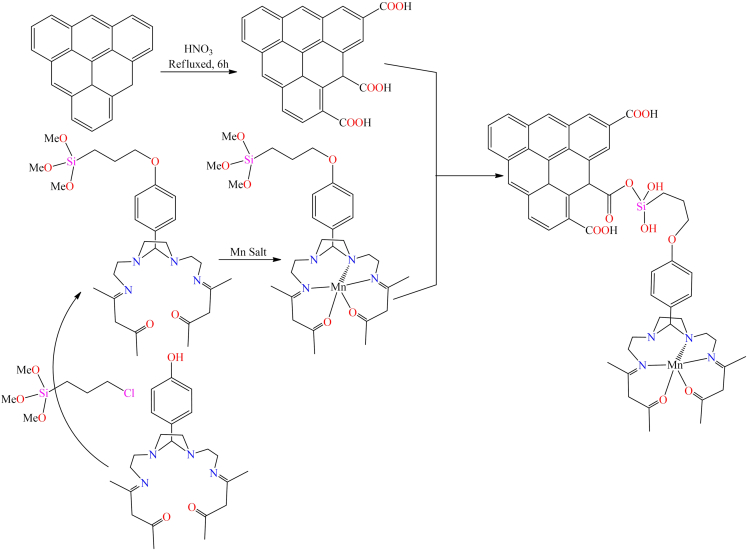
Heterogeneous catalyst Mn(II)-L@PCox prepared by forming covalent linkages between oxidized pyrolytic carbon (PCox) and a metal-silane precursor complex (Mn(II)-L-OS) Redrawn based on ref.^132^

However, in 2015, a highly efficient and innovative heterogeneous Mn-RC catalyst was developed using recycled materials, specifically pyrolytic carbon derived from tires.^132^ This study demonstrated a proof-of-concept for synthesizing the anchored catalyst Mn^II^L@PCox by covalently bonding a Mn(II) complex to the PCox support material (As shown in Scheme 2). The Mn^II^L@PCox heterogeneous catalyst was utilized for the degradation of the dye methyl orange in water (H_2_O). A comparative study with the homologous Mn(II)-L@ACox counterpart revealed that PCox provides a more stable matrix, effectively protecting the active Mn centers from oxidative degradation. This stability allows the catalyst to maintain rapid activity even under high oxidation potentials. Furthermore, the carbon framework offers a compact structure in which all Mn complexes are well-exposed to the reaction medium, thereby minimizing potential kinetic limitations. The findings concluded that PCox provides a stable and compact matrix, effectively exposing all active manganese centers, thereby enhancing the catalyst’s performance in dye degradation.

**Scheme 3:**
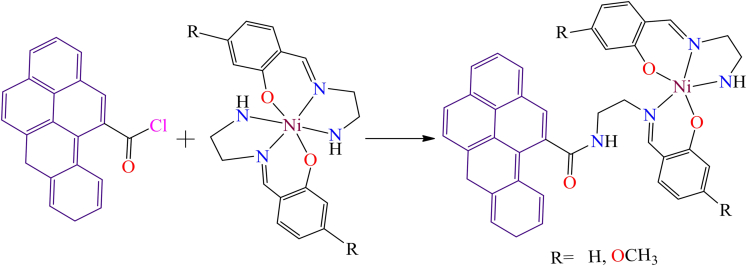
Scheme represents the heterogenization of homogenous Ni-complexes on functionalized activated carbons^85^

**Scheme 4:**
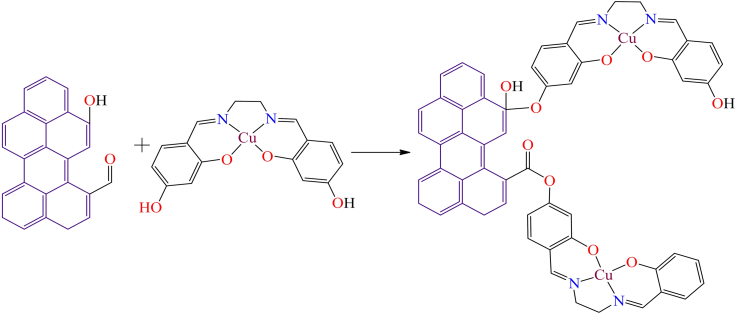
Immobilization of copper(II) Schiff base complex [Cu(4-HOsalen)] on the surface of oxidised activated carbon Redrawn based on ref.^134^

A.R. Silva et al.^133^ reported two Schiff base nickel(II) complexes: bis[4-methoxy-o-[N-(3-aminopropyl)formimidoyl]phenolato-O,N,N']-nickel(II) and bis[o-[N-(3-aminopropyl) formimidoyl]phenolato-O,N,N']-nickel(II). Both nickel complexes contain amine groups, which facilitate their anchoring onto chemically oxidized activated carbon treated with thionyl chloride. The immobilization process involves two sequential steps: 1. functionalization of activated carbon (The activated carbon is first oxidized using nitric acid, followed by treatment with thionyl chloride to introduce acyl chloride groups), and 2. Anchoring of nickel complexes (The amine groups in the nickel complexes react with the acyl chloride-functionalized activated carbon, leading to stable immobilization (as illustrated in Scheme 3)). Additionally, in 2002, A.R. Silva et al. synthesized a copper(II) Schiff base complex [Cu(4-HOsalen)], which contains hydroxyl groups that enable direct anchoring onto the surface of air-oxidized activated carbon (Scheme 4).^134^ The physical and chemical properties of the complex were analyzed using various spectroscopic and characterization techniques, including UV-Vis spectroscopy, SEM, X-ray photoelectron spectroscopy (XPS), nitrogen adsorption isotherms, EPR, and thermal analysis.

**Figure 2:**
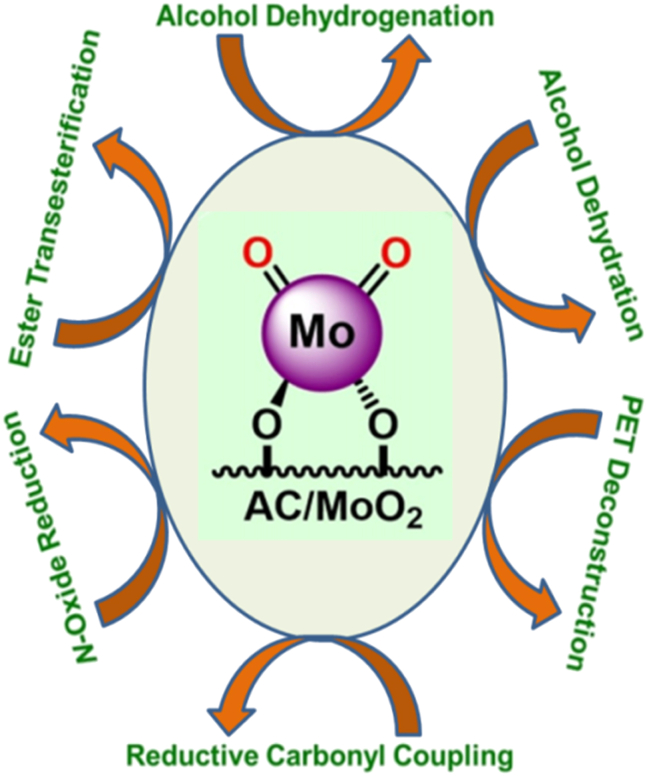
Carbon supported MoO_2_ species as a single site heterogeneous catalyst for various organic transformation reactions

Although, Yiqi Liu et al. published a mini-review on single-site carbon-supported oxo-metal complexes.^135^ Their study focused on carbon-grafted molybdenum dioxo species as a single-site heterogeneous catalyst for various chemical transformations (Figure 2). In this mini-review, the authors discuss the catalytic cycles of various organic transformations mediated by single-site, carbon-supported oxo-metal complexes. Looking forward, such single-site catalytic systems are anticipated to gain prominence as highly desirable and efficient platforms for future applications.

## Carbon nanotubes as solid supports in heterogeneous catalysis

Carbon nanotubes (CNTs) are allotropes of carbon with a one-dimensional tubular or cylindrical nanostructure, featuring diameters in the nanoscale range. These nanotubes exhibit exceptional thermal conductivity,^136^^,^^137^ remarkable mechanical strength,^138^^,^^139^^,^^140^ high tensile strength, and excellent electrical properties,^141^ CNTs are commonly classified into two types for use as solid supports in heterogeneous catalysis: single-walled carbon nanotubes (SWCNTs) and multi-walled carbon nanotubes (MWCNTs). Catalysts grafted onto carbon nanotubes are gaining attention as a new generation of heterogeneous catalysts due to their enhanced stability, surface area, and reactivity. CNT-grafted catalysts are gaining recognition as a promising new generation of heterogeneous catalysts. Due to their exceptional chemical and mechanical stability, as well as their high surface area, carbon nanotubes are regarded as an excellent choice in various fields, including electronics,^142^ polymer composites,^143^ optics,^144^ and, most notably, catalysis.^145^^,^^146^^,^^147^

## Single walled carbon nanotube supported catalysis

**Figure 3:**
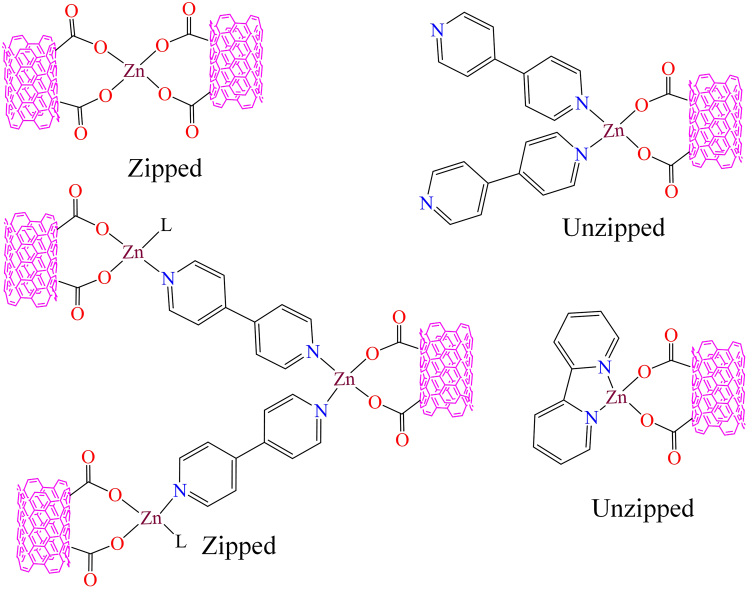
Unzipped and zipped complexes of Zink metal with SWCNT Redrawn based on ref.^149^

Single-walled carbon nanotubes (SWNTs) have significant attention for the heterogenization of homogeneous complexes due to their unique properties. Accordingly, In 2009, Eden W. McQueen et al. reported the anchoring of Co(II) complexes onto SWNTs and examined their electrochemical properties.^148^ However, Lingjie Meng et al. developed a zinc complex based on single-walled carbon nanotubes using a relatively simple, cost-effective method.^149^ During synthesis, they reacted carboxylic acid-functionalized SWCNTs with Zn(OAc)_2_, followed by the addition of *2,2′*-bipyridine or *4,4′*-bipyridine. In the initial step, the carboxylic acid-functionalized SWCNTs formed a complex with the zinc metal unit. The researchers described two possible products with the ligands *2,2′*-bipyridine or *4,4′*-bipyridine: one with the zinc unit “zipped” and the other “unzipped” (as shown in Figure 3).

**Figure 4:**
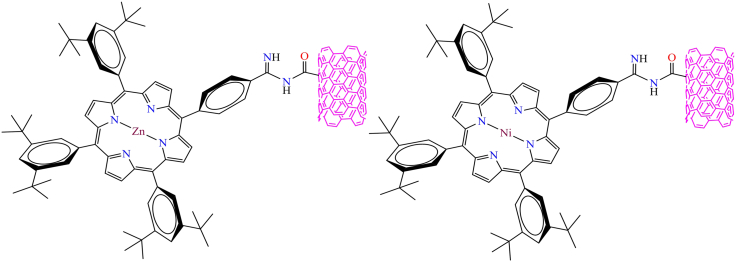
Molecular Structures of Supramolecule with SWCNT^150^ Copyright © 2017, ACS.

However, in 2017, Laura Rodríguez-Perez et al. demonstrated the electronic interaction between a novel metal-porphyrin (supramolecular) and oxidized single-walled carbon nanotubes through amidinium-carboxylate connectivity (as shown in Figure 4).^150^ The authors synthesized novel compounds through a Tour reaction between functionalized SWCNTs and benzoic acid moieties. Their investigations revealed strong electronic interactions between carboxylate-amidinium pairs. Furthermore, a combination of techniques, including XPS, TGA, UV-Vis, Raman, and TEM, was employed to characterize the physicochemical properties of the system.

**Figure 5:**
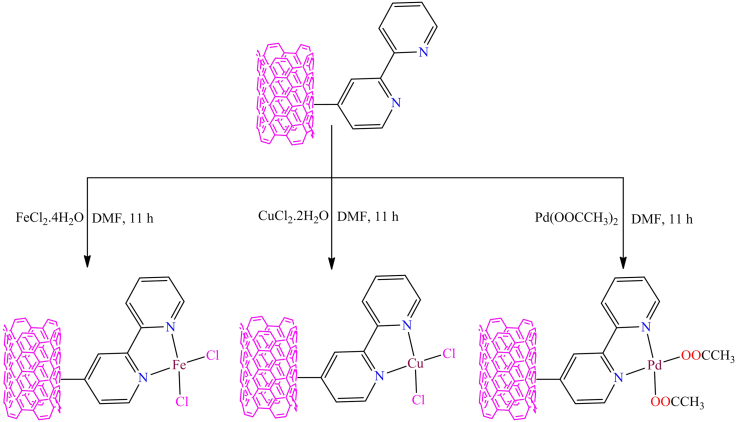
Synthesis of single walled carbon-nano hybrids Redrawn based on ref.^151^

Although, Magdalena Nosek et al. synthesized *2,2′*-bipyridine-functionalized single-walled carbon nanotubes.^151^ The bipyridine-functionalized SWCNTs serve as an organic framework for various transition metals, including copper, iron, and palladium. They also investigated the charge transfer between the metal and the modified SWCNTs, either from the metal to the nanotube or vice versa. The synthesis process was carried out in two steps: (1) preparation of the *2,2′*-bipyridine-functionalized SWCNTs, and (2) reaction with transition metal salts to form heterogeneous metal complexes with copper, iron, and palladium (Figure 5). The compounds were characterized using several techniques, including FT-IR, XPS, and Raman spectroscopy.

**Figure 6:**
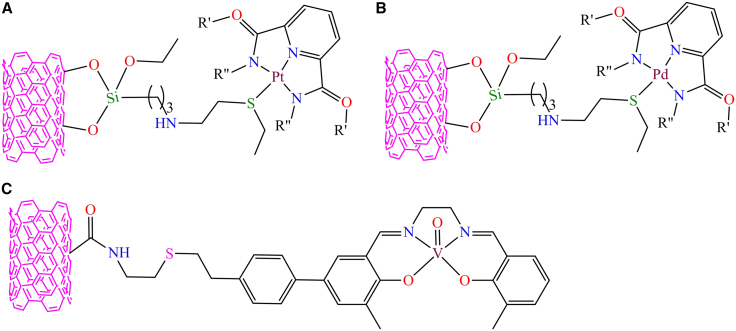
Single-walled carbon nanotubes composite of pincer Pt/Pd complexes^152^^,^^153^ (A and B) SWCNT-based composites of pincer Pt/Pd complexes for the oxidation of cyclohexane with O₂ under solvent-free conditions. (C) SWCNT-supported oxo-vanadium Schiff base complex for the catalytic cyanosilylation of aldehydes. Copyright © 2004, Elsevier.

The arc discharge technique was employed to synthesize single-walled carbon nanotube composites containing pincer Pt/Pd complexes through covalent immobilization.^152^ Initially, *3-*aminophenyl trimethyl oxysilane-modified SWCNTs were used as a benchmark for the Pt/Pd complexes, as shown in Figures 6A and 6B). These supported complexes functioned as efficient nanocatalysts for cyclohexane oxidation with O_2_ under solvent-free conditions, achieving turnover numbers (TON) ranging from 1678 to 1946. The SWNTs/Pd catalyst exhibited 22.7% conversion with 93.3% selectivity, while the SWNTs/Pt system showed 20.7% conversion with 95.1% selectivity. This suggests that the SWNTs/Pd and SWNTs/Pt nanocatalyst excels in terms of turnover numbers and selectivity. However, a single-walled carbon nanotube-supported oxo-vanadium Schiff base complex was used for the cyanosilylation of aldehydes as shown in Figure 6C.^153^ The authors utilized mercapto-modified SWCNTs as supports for the covalent attachment of a styryl-functionalized vanadyl Schiff base complex to create a heterogeneous catalyst. This anchoring occurred through a radical chain mechanism. The authors reported that the supported SWNT-vanadyl-modified solid catalyst achieved a 95% conversion, whereas the unsupported catalyst showed only 83% conversion under comparable conditions. The reactions were carried out using substrate (0.41 mmol), TMSCN (3 equivalents), catalyst (0.3 mol %), nitrobenzene (0.41 mmol), and CHCl_3_ (0.5 mL) at room temperature under a nitrogen atmosphere for 12 h. SWNT-vanadyl-modified solid acted as a reusable heterogeneous catalyst for the efficient cyanosilylation of aldehydes with trimethylsilylcyanide. No leaching was observed in this system, ensuring that there was no loss of activity, thus confirming its function as a true heterogeneous catalyst.

## Multi walled carbon nanotube supported catalysis

**Scheme 5:**
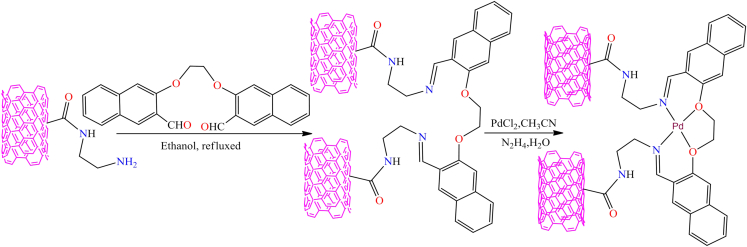
Synthesis of a novel Schiff base-MWCNTs-Pd nanocatalyst Redrawn based on ref.^154^

Multi-walled carbon nanotubes (MWCNTs) combined with Schiff base-functionalized metal nanoparticles serve as important nanocatalysts for organic transformation reactions. In 2017, palladium metal was incorporated into novel Schiff base-functionalized MWCNTs.^154^ The synthesis of the Schiff base-MWCNTs-Pd nanocatalyst involved the immobilization of Schiff base onto amine-functionalized MWCNTs through covalent grafting, followed by reaction with palladium salt (as shown in Scheme 5). This Schiff base-MWCNTs-Pd nanocatalyst was used for the Suzuki-Miyaura cross-coupling reaction under mild conditions. To obtain the maximum product yield (approximate 96%), the authors systematically optimized the reaction conditions and established the ideal parameters as follows: 0.2 mol % of the Pd-based nanocatalyst, 1.0 mmol of 4-bromotoluene, 1.0 mmol of phenylboronic acid, 2.0 mmol of base, and 3 mL of solvent at room temperature. They also tested the reusability of the Schiff base-MWCNTs-Pd nanocatalyst over four cycles and observed minimal loss of activity, confirming the catalyst’s excellent performance as a heterogeneous catalyst.

**Scheme 6:**
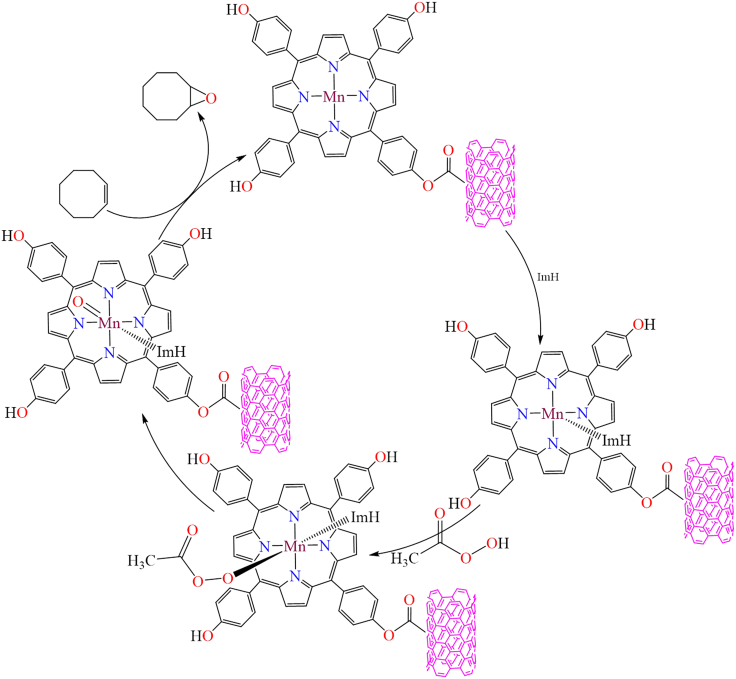
Proposed mechanism for alkene oxidation with manganese-porphyrin based heterogeneous catalysts [Mn(THPP)OAc@MWCNT] Redrawn based on ref.^155^

Moreover, in 2016, a manganese-porphyrin-based reusable heterogeneous catalyst, [Mn(THPP)OAc@MWCNT], was reported for the oxidation of various alkenes and alkanes using urea hydrogen peroxide (UHP).^155^ The functionalized multi-walled carbon nanotubes were employed as a solid support for immobilizing a meso-tetrakis(*4*-hydroxyphenyl)porphyrin-based manganese complex, which was characterized through elemental analysis, FT-IR, UV-Vis, powder XRD, and SEM. This heterogeneous catalyst was employed for the oxidation of various alkenes and achieved up to 100% conversion under specific reaction conditions, namely: 0.3 mmol of substrate, 0.003 mmol of catalyst, 0.15 mmol of imidazole, 0.75 mmol of urea-hydrogen peroxide (UHP), 1.8 mmol of acetic anhydride, and 1 mL of solvent. Scheme 6 illustrates the proposed mechanism for alkene oxidation. During the catalytic process, acetic anhydride acted as an oxidant activator, used in stoichiometric amounts. Moreover, this catalyst could be reused multiple times without any loss in catalytic activity. The authors concluded that the manganese-porphyrin-based heterogeneous catalyst [Mn(THPP)OAc@MWCNT] was highly efficient and reusable for the epoxidation of alkenes and oxidation of alkenes using UHP.

**Scheme 7:**
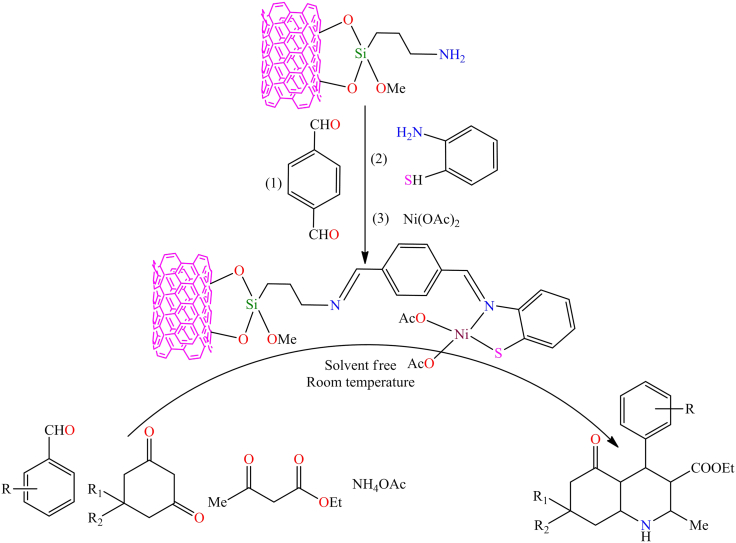
Synthesis of polyhydroquinoline derivatives through Hantzsch condensation reaction (four-component condensation) using immobilized nickel(II) Schiff-base complex supported on MWCNTs (catalyst (0.005 g), aromatic aldehyde (1 mmol), 1,3-dione (1 mmol), ethyl acetoacetate (1 mmol), and ammonium acetate (1.5 mmol) in solvent-free at RT)

**Figure 7:**
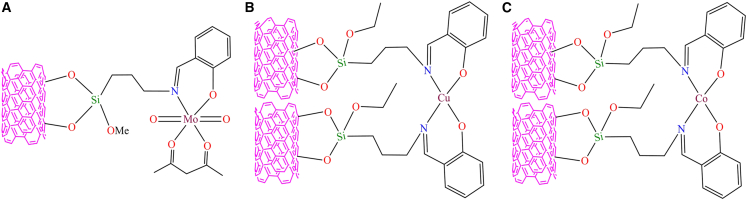
Schiff base metal complexes immobilised on modified MWCNTs (A) Mo-Schiff base complex. (B) Cu-Schiff base complexes. (C) Co-Schiff base complexes. Redrawn based on ref.^156^^,^^157^

Although, Jamshid Rakhtshah et al. synthesized a green and efficient heterogeneous nickel(II) Schiff-base complex that acts as a catalyst for the Hantzsch one-pot condensation reaction.^156^ The synthesis of the supported Ni complex involved several steps. First, multi-walled carbon nanotubes (MWCNTs) were oxidized and then reacted with *3-*aminopropyltrimethoxysilane (APTMS) to form APTMS-modified MWCNTs. These modified CNTs, with -NH_2_ functional groups, were then reacted with terephthalaldehyde and *2-*aminothiophenol to create an organic framework that coordinates with Ni metal, as shown in Scheme 7. The study demonstrated that the Ni-based heterogeneous catalyst is economical, efficient, and offering high product yields, shorter reaction times, a cleaner reaction pathway, and excellent reusability. Notably, only 0.005 g of the catalyst enabled the synthesis of polyhydroquinoline derivatives with an approximate yield of 95% via the Hantzsch one-pot condensation of aromatic aldehydes (1 mmol), *1,3*-diones (1 mmol), ethyl acetoacetate (1 mmol), and ammonium acetate (1.5 mmol) at room temperature under solvent-free conditions. Additionally, a new molybdenum-Schiff base complex was effectively dispersed on the surface of multi-walled carbon nanotubes through covalent bonding.^157^ Figure 7A illustrates the immobilized molybdenum-Schiff base complex, MoO_2_(acac)sal-MWCNTs, synthesized from APTMS-modified MWCNTs. The resulting MoO_2_(acac)sal-MWCNTs nanomaterial exhibited excellent stability and catalytic performance in the epoxidation of olefins, achieving up to 99% conversion and 99% selectivity in the presence of *tert*-butyl hydroperoxide (TBHP) and cumene hydroperoxide (CHP) under mild reaction conditions (100 mg catalyst, 8 mmol olefin, 14.4 mmol TBHP, and 10 mL chloroform).

**Figure 8:**
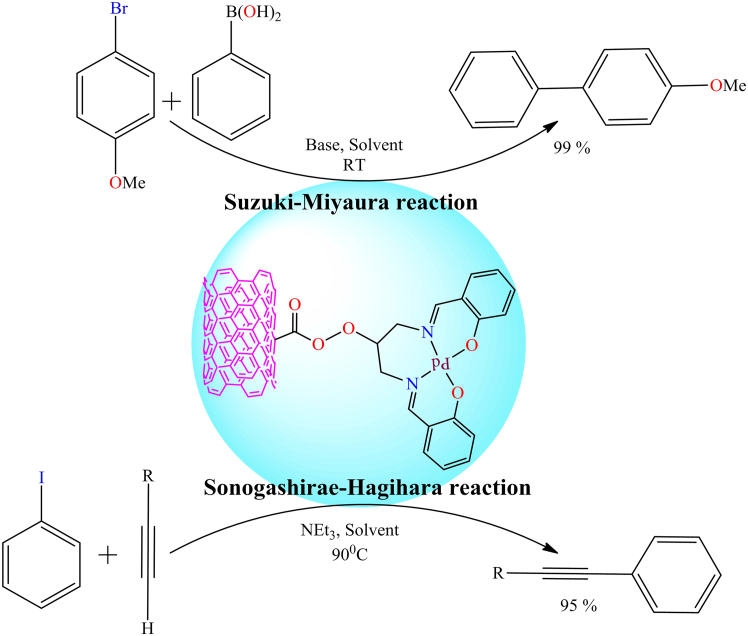
Pd-catalysts (Pd-Schiff base@MWCNTs) and its activity toward Suzuki-Miyaura coupling and Sonogashirae-Hagihara reaction

However, Lu Bai et al. developed novel metal Schiff base complexes immobilized on APTMS-modified multi-walled carbon nanotubes.^158^ They used the modified MWCNTs to immobilize the Schiff base, followed by reaction with copper and cobalt ions to form heterogeneous copper and cobalt Schiff base complexes (Figures 7B and 7C). These heterogeneous complexes serve as highly efficient electrocatalysts for the oxygen reduction reaction. While pristine MWCNTs possess minimal catalytic activity, their incorporation into hybrid heterogeneous systems significantly improves ORR performance in alkaline media, facilitating a complete four-electron reduction pathway at metal-free active sites. This improvement is primarily attributed to the synergistic interaction between metal Schiff-base complexes and the MWCNT framework. Additionally, Mozhgan Navidi et al. synthesized an efficient heterogeneous palladium catalyst.^159^ They used multi-walled carbon nanotubes as a solid support for the covalent anchoring of palladium Schiff base complexes, resulting in an air and moisture-stable, recyclable palladium(II) Schiff base complex (Pd-Schiff base@MWCNTs). Figure 8 illustrates the Pd-catalyst and its activity in the Suzuki-Miyaura coupling of aryl halides and arylboronic acids, as well as the Sonogashira-Hagihara reaction of terminal alkynes and aryl iodides. The supported catalyst delivered product yields of up to 99% in the Suzuki-Miyaura coupling and around 95% in the Sonogashira-Hagihara reaction. Moreover, the Pd-based catalyst exhibited remarkable thermal stability, oxygen tolerance, recyclability, and high catalytic activity. Its straightforward preparation and facile separation from the reaction mixture further highlight its potential as an efficient heterogeneous catalytic system.

**Scheme 8:**
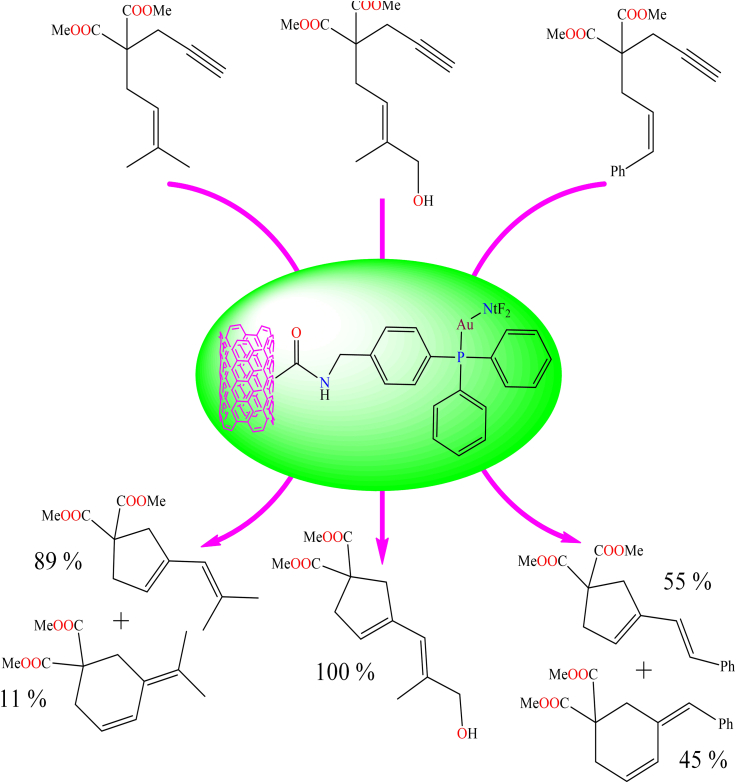
Gold complex supported heterogeneous catalyst for cyclization of *1,6* enynes

However, Deborah Brazzolotto et al. developed a series of bis(*2,9*-dialkyl-*1,10*-phenanthroline) copper complexes for immobilization on multi-walled carbon nanotubes (MWCNTs).^160^ They designed and synthesized new ligands, including *2*-(*5*-(pyren-*1*-yl)pentyl)-*9*-methyl-*1,10*-phenanthroline, along with their corresponding copper complexes, bis(*2,9*-dialkyl-*1,10*-phenanthroline) copper(II) complexes, which efficiently undergo autoreduction to copper(I). These MWCNT-supported complexes exhibited a low overpotential for the 4H^+^/4e^−^ oxygen reduction reaction at pH 5, with a potential of 0.86 V. However, a gold complex, [Tf_2_NAuPPh_3_CH_2_NH_2_], was covalently anchored onto functionalized multi-walled carbon nanotubes, which were used as nanocatalysts for cyclization reactions.^161^ Scheme 8 illustrates the gold-supported heterogeneous catalysts used in the cyclization of *1,6*-enynes, such as dimethyl *2-*(*3*-methylbut-*2*-en-*1*-yl)-*2*-(prop-*2*-yn-*1*-yl)malonate, (E)-dimethyl *2*-(*4*-hydroxy-*3*-methylbut-*2*-en-*1*-yl)-*2*-(prop-*2*-yn-*1*-yl)malonate, and (Z)-dimethyl *2*-(*3*-phenylallyl)-*2*-(prop-*2*-yn-*1*-yl)malonate. During the cyclization process, five-membered products were the major products, while six-membered products were formed as the minor products (as shown in Scheme 8). The activity and recyclability of the heterogeneous catalyst were significantly improved compared to their homogeneous counterparts.

**Figure 9:**
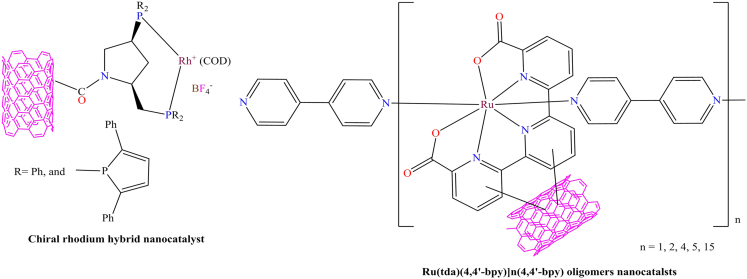
Chiral rhodium hybrid nanocatalysts and ruthenium oligomer nanocatalyst Redrawn based on ref.^162^^,^^163^

Moreover, C. C. Gheorghiu et al. and M. A. Hoque et al. developed chiral rhodium hybrid nanocatalysts and ruthenium oligomer nanocatalysts, with their reports published in Dalton Trans and Nature Chemistry in 2014 and 2020, respectively.^162^^,^^163^ The chiral rhodium nanocatalysts were prepared by covalently linking functionalized carbon nanotubes, as shown in Figure 9. These nanocatalysts displayed excellent catalytic performance, achieving up to 100% conversion and enantioselectivity as high as 99% in the asymmetric hydrogenation of methyl *2-*acetamidoacrylate and *α-*acetamidocinnamic acid. The reactions were carried out under comparable conditions, employing 80 mg of substrate, 30 mg of catalyst, 5.5 bar of H_2_ pressure, and 7 mL of anhydrous methanol. In contrast, the ruthenium-based oligomeric complex was heterogenized via CH-π interactions, wherein the ruthenium-bound ligand interacted with the hexagonal aromatic rings on the surface of multi-walled carbon nanotubes (MWCNTs), as illustrated in Figure 9. The resulting heterogeneous materials functioned as electroanodes, efficiently catalyzing water oxidation at neutral pH (pH 7) with high current density. Furthermore, the authors examined the anchoring strategy based on aromatic CH-π interactions and demonstrated that this approach provides a robust, convenient, and efficient method for immobilizing ligands onto CNT surfaces. Importantly, both nanocatalysts exhibited excellent reusability, retaining their catalytic activity over multiple cycles, thereby confirming their potential as recyclable nanocatalysts.

## Graphene oxid (GO) as solid supports in heterogeneous catalysis

Graphene is an allotrope of carbon with a two-dimensional arrangement of atoms in a single sheet, resembling a honeycomb structure.^164^^,^^165^ Initially, it was identified as a single-layered sheet-like material. Graphene undergoes oxidation to form graphene oxide, which contains oxygenated functional groups such as hydroxyl, epoxy, and carboxylic acid.^166^ These functional groups facilitate the grafting or immobilization of catalytically active components.^167^^,^^168^^,^^169^ Due to its high thermal stability, large surface area, chemical durability, and intrinsic reactivity, graphene oxide serves as an effective solid support for various metal complexes.^170^ Additionally, numerous catalytically active metals (such as gold, silver, palladium, platinum, nickel, copper, ruthenium, and rhodium) and metal oxides (including TiO_2_, ZnO, SnO_2_, MnO_2_, Co_3_O_4_, Fe_3_O_4_, NiO, Cu_2_O, RuO_2_, and SiO_2_) have been reported in the literature as being immobilized on the nanoscale surface of graphene oxide.^171^^,^^172^^,^^173^^,^^174^^,^^175^^,^^176^^,^^177^^,^^178^^,^^179^ However, this review primarily focuses on the immobilization of homogeneous complexes onto graphene oxide sheets and their applications in various fields, including organic transformation reactions.

## Non-modified graphene oxide as solid supports in catalysis

**Figure 10:**
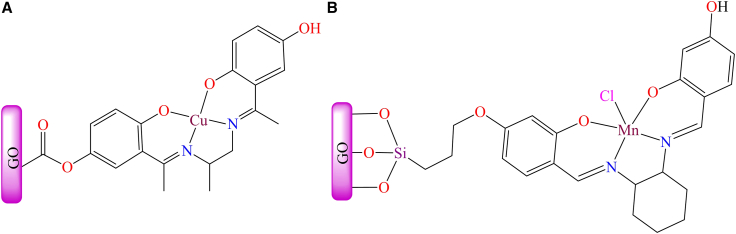
Graphene-anchored Schiff base metal complexes (A) GO supported copper complex [RGO-CuL]. (B) Mn-based complex supported on *3*-chloropropyltrimethoxysilane modified GO sheet. Redrawn based on ref.^180^^,^^181^

Unmodified graphene oxide (GO) has been employed as a solid support for anchoring metal complexes. Due to its exceptional mechanical strength, high surface area, electrical conductivity, and chemical stability, non-modified graphene can serve directly as a solid support in various applications. Figure 10A depicts a graphene-anchored copper Schiff base complex.^180^ The complex was synthesized using a tetradentate Schiff base ligand containing N,N,O,O donor atoms, which reacted with a copper salt, followed by interaction with graphene oxide nanosheets to form RGO-CuL. The graphene oxide-supported copper complex, RGO-CuL, served as a heterogeneous catalyst for the oxidation of alkenes in the presence of hydrogen peroxide, achieving a turnover number (TON) of 47,000, the highest reported at that time. The reaction was carried out under the conditions: catalyst: alkene: H_2_O_2_ = 1:50:500 in ethanol at 50°C for 1 h. The catalyst demonstrated excellent recyclability, allowing it to be used multiple times for the efficient oxidation of alkenes.

**Figure 11:**
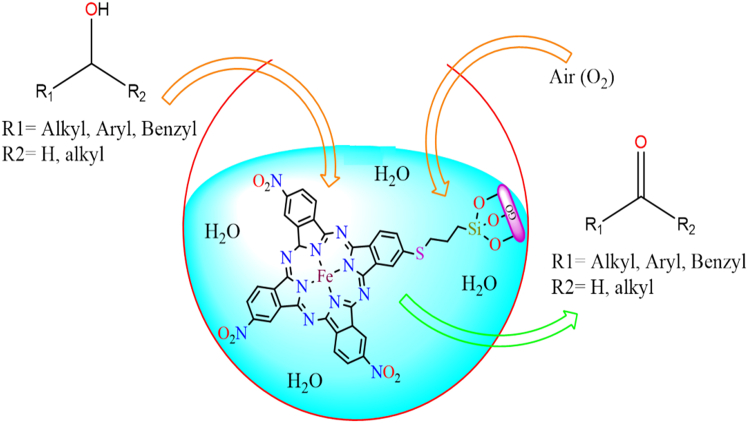
Oxidation of various alcohols in presence of water and air by graphene oxide supported iron phthalocyanine complex

Moreover, in 2014, a chiral magnesium complex was reported in the literature,^181^ which was anchored to the surface of a *3*-chloropropyltrimethoxysilane-modified graphene oxide sheet through a covalent bond (as shown in Figure 10B). The supported chiral Mn-based heterogeneous catalyst effectively catalyzed the enantioselective epoxidation of a broad range of alkenes, achieving conversions of up to 96% under the optimized conditions: 1 mmol of substrate, 3 mL of dichloromethane (DCM), 2 mmol of oxidant, and 2 mol % of catalyst. The heterogeneous catalyst exhibited high catalytic efficiency, reusability, and superior selectivity compared to its homogeneous counterpart. Although, iron phthalocyanine complex was synthesized using *P*-NO_2_ phthalimide anhydride, urea, FeCl_3_, and ammonium molybdate through a template method in a microwave oven. It was then reacted with *3*-(trimethoxysilyl)propane-*1*-thiol under a nitrogen atmosphere to form the tetranitro iron phthalocyanine complex, which was immobilized onto the surface of graphene oxide sheets.^182^ The graphene-supported iron phthalocyanine-based heterogeneous catalysts actively participated in the selective oxidation of various alcohols to their corresponding aldehydes and ketones in the presence of air (as shown in Figure 11). This catalytic process was environmentally friendly, utilizing water as the solvent and air as the oxidant. The catalyst achieved up to 98% product yield under comparable reaction conditions, using 1.0 mmol of alcohol, 0.5 mmol of K_2_CO_3_, 0.4 mol % of catalyst, 4.0 mL of water, at 1 atm oxygen pressure, and a temperature of 60°C. Additionally, the easy separation of products and catalysts made the process highly feasible.

**Figure 12:**
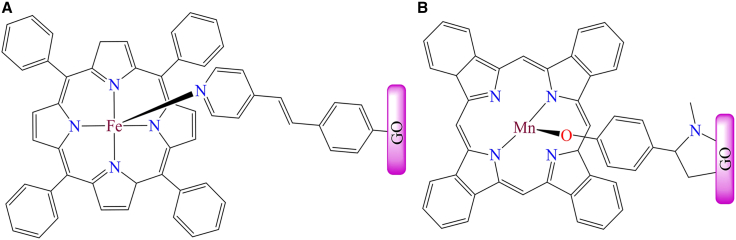
Modified graphene-supported porphyrin based complexes (A) Iron porphyrine complex (B) Manganese phthalocyanine complex.^183^^,^^184^ Copyright © 2012, ACS.

Additionally, an iron porphyrin complex was immobilized on pyridine-functionalized graphene oxide and used for oxygen reduction reactions (ORR).^183^ Figure 12A illustrates the graphene-supported iron porphyrin complex, which functions as a four-electron transfer catalyst for ORR and can be utilized in Pt-free cathodes for alkaline direct methanol fuel cells. Although, Figure 12B shows a manganese phthalocyanine complex anchored to the surface of graphene oxide through covalent linkages.^184^ This heterogeneous catalyst functions as a photocatalyst, facilitating intermolecular electron transfer reactions for molecular hydrogen production. The nanocomposite achieved hydrogen yields of 8.59 and 1.45 mmol mg^−1^ under 10 h of UV-Vis and visible light (400 nm) irradiation, respectively. The material was characterized using SEM, TEM, UV-Vis, FTIR, and Raman spectroscopy.

**Figure 13:**
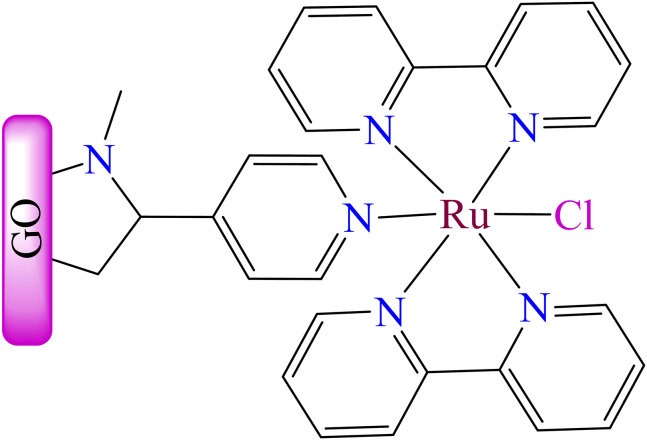
Graphene oxide supported ruthenium complex [Ru(bpy)_2_(py)]Cl^185^ Copyright © 2013, ACS.

Moreover, B. Xiao et al. developed a novel ruthenium bipyridine-based nanocomposite for dye absorption.^185^ They synthesized the ruthenium bipyridine complex, ((*2,2′*-bipyridyl)-*4*-pyridyl-chlororuthenium(II) [Ru(bpy)_2_(py)]Cl), which was covalently anchored to graphene sheets through a *1,3*-dipolar cycloaddition of azomethine ylides (as shown in Figure 13). As a result, a rapid and coordinated photo-induced electron transfer reaction occurred between the graphene and [Ru(bpy)_2_(py)]Cl complex. The graphene oxide-supported ruthenium complex acted as a photocatalyst for hydrogen evolution from water under UV-Vis light irradiation. Under 10 h of UV-Vis or visible light irradiation (400 nm), the Ru(bpy)_2_(py)Cl/G/Pt nanohybrid produced 39.3 and 7.6 μmol mg^−1^ of H_2_, respectively. The catalyst also exhibited excellent stability, maintaining nearly constant hydrogen yields over 50 h of continuous irradiation. These findings indicate that the carbon-based nanohybrid, composed of organic dye molecules covalently functionalized with graphene, is a promising photocatalyst for efficient hydrogen evolution.

## Modified graphene oxid as solid supports in catalysis

**Scheme 9:**
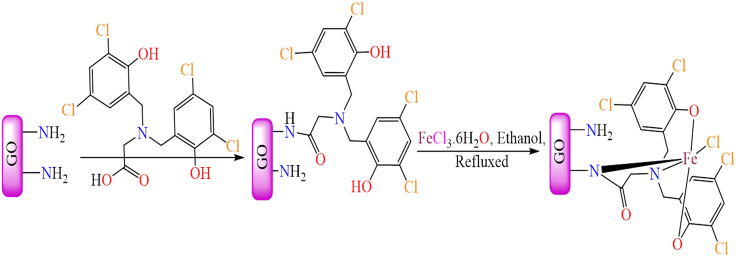
Synthetic procedure of graphene oxide supported iron complex (FeLGDC-AP@GO) Redrawn based on ref.^186^

In addition to non-modified graphene oxide, modified graphene oxide has also been utilized as a solid support for metal complexes. Graphene oxide modified with *3-*aminopropyltriethoxysilane (APTES) introduces -NH_2_ functional groups on its surface, creating reactive sites for the immobilization of metal complexes. As a result, various transition metal Schiff base complexes have been anchored onto APTES-modified graphene oxide and utilized as catalysts in diverse organic transformation reactions. A glycine-derived bis(phenol) amine ligand was synthesized and immobilized onto the surface of APTES-modified graphene. This functionalized graphene then underwent metalation with an iron salt, forming a heterogeneous iron complex (FeLGDC-AP@GO), as illustrated in Scheme 9.^186^ The synthesis involved two key steps: First, amine-functionalized graphene oxide (GO) was prepared by reacting graphene oxide (GO) with *3*-aminopropyltriethoxysilane. In the second step, a heterogenized ligand was synthesized by covalently bonding the modified graphene oxide with [*2*-(bis(*3,5*-dichloro-*2*-hydroxybenzyl)amino) acetic acid], followed by metalation with an iron salt to obtain the heterogeneous iron complex (FeLGDC-AP@GO). The GO-supported iron complex acted as a highly efficient catalyst for the selective oxidation of sulfides, achieving up to 100% conversion with complete (100%) selectivity toward sulfoxides under the optimized reaction conditions: 1 mmol of sulfide, 2 equivalents of H_2_O_2_, 1 mol % of the supported catalyst, and a 1:1 H_2_O-acetone mixture (2 mL) at room temperature. Moreover, the catalyst exhibited excellent recyclability, retaining its high activity and selectivity during successive cycles of sulfide oxidation to the corresponding sulfoxides under similar conditions. This catalytic approach was environmentally friendly, as it avoided the use of hazardous chemicals, making it a greener alternative for oxidation reactions.

**Figure 14:**
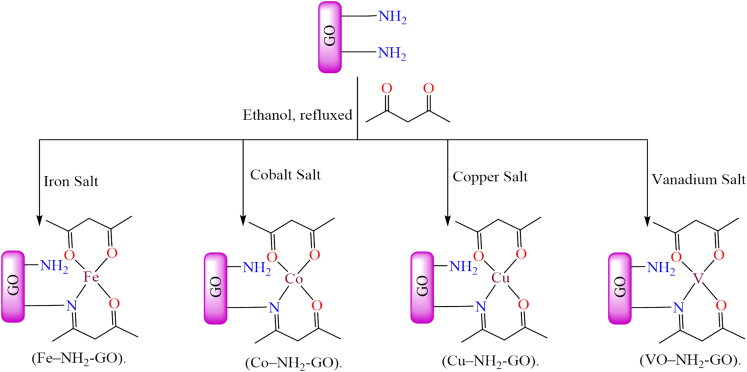
Various graphene oxide supported transition metal (Fe^2+^, Co^2+^, VO^2+^, or Cu^2+^) Schiff base complexes Redrawn based on ref.^187^

However, Figure 14 illustrates various transition metal Schiff base complexes immobilized on the nanoscale surface of graphene oxide.^187^ In this anchoring process, *3*-aminopropyltriethoxysilane (APTES)-modified GO was utilized as a solid support for the attachment of Fe^2+^, Co^2+^, VO^2+^, and Cu^2+^ Schiff base complexes. These GO-supported metal complexes were employed as catalysts for the epoxidation of various alkenes. Among the tested catalysts; Co (12%), Fe (30%), and V (31%), the Cu-supported complex demonstrated the best performance, delivering more than 94% conversion and 99% selectivity under optimized conditions (catalyst: 10 mg; styrene: 0.67 mL; styrene-to-oxidant ratio: 1:3; solvent: 5 mL CH_3_CN; temperature: 80°C; reaction time: 7 h). During the epoxidation reaction, the active catalytic species was identified as a metal-peroxo intermediate, formed via interaction with *tert*-butyl hydroperoxide. Furthermore, the supported catalyst displayed excellent stability and could be reused for up to four consecutive cycles without any significant decline in activity.

**Scheme 10:**
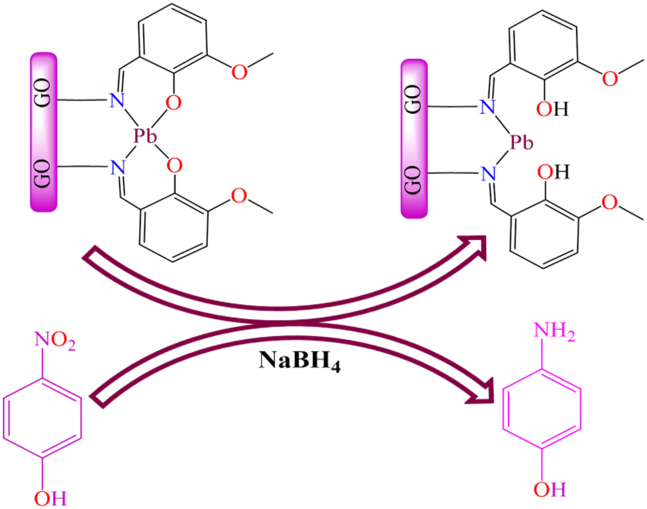
Magnetic heterogeneous catalysts for the reduction of *p*-nitrophenol in presence of sodium borohdride (NaBH_4_) (1 mM *p-*nitrophenol (200 μL), 0.1 M NaBH4 (200 μL), deionized water (2.5 mL), GO-MnFe_2_O_4_ (100 μL of 1.4 mg mL^−1^))

Although, in 2018, K. Karami et al. synthesized a graphene-immobilized palladium Schiff base complex and reported their findings in Dalton transactions.^188^ They used *3*-aminopropyltriethoxysilane (APTES)-modified magnetic GO-MnFe_2_O_4_ as a benchmark solid support, which was covalently bonded to the ligand and subsequently metalated to form a graphene oxide-supported palladium complex. These nanohybrid materials were employed as magnetic heterogeneous catalysts for the reduction of *p*-nitrophenol in the presence of sodium borohydride (NaBH_4_), as depicted in Scheme 10. The supported catalyst achieved 100% yield of *p*-aminophenol under the reaction conditions: 1 mM *p*-nitrophenol (200 μL), 0.1 M NaBH_4_ (200 μL), deionized water (2.5 mL), and GO-MnFe_2_O_4_ (100 μL of 1.4 mg mL^−1^). The authors tested the catalyst over five consecutive cycles and observed no significant loss in activity or selectivity, confirming its reusability as an efficient catalytic system.

**Figure 15:**
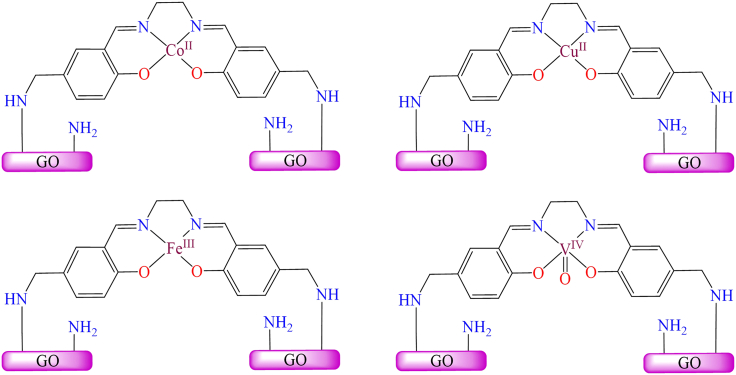
Cu(II) and Co(II) salen complexes immobilized on modified GO Redrawn based on ref.^189^^,^^190^

Additionally, Zhifang Li et al. synthesized Cu(II), Co(II), Fe(III), and V(IV) salen complexes (as shown in Figure 15) and immobilized them onto APTES-modified graphene oxide (GO) nanosheets.^189^^,^^190^ The immobilization process involved two key steps: First, graphene oxide was functionalized with amine (-NH_2_) groups through its reaction with *3*-aminopropyltriethoxysilane (APTES). This was followed by the covalent attachment of CM-salen ligands and subsequent coordination with metal salts to form the desired complexes. The synthesized GO-supported metal complexes were employed as heterogeneous oxidation catalysts for the oxidation of styrene, using isobutyraldehyde as a co-reductant and air as the oxidant in acetonitrile solvent. Under identical reaction conditions (catalyst: 50 mg; styrene: 10 mmol; solvent: 10 mL CH_3_CN; co-oxidant: 25 mmol isobutyraldehyde; temperature: 80°C; reaction time: 6 h), the supported cobalt-salen catalyst afforded 70.1% styrene conversion, while the copper-salen analogue achieved 73.5% conversion. In comparison, the iron- and vanadium-immobilized catalysts exhibited higher activity, reaching 76.5% and 88.0% conversion, respectively. The authors also investigated the reaction mechanism and identified metal peroxo species as the key active intermediates in the catalytic cycle. These heterogeneous catalysts demonstrated high reactivity, selectivity, and excellent reusability over multiple cycles.

**Scheme 11:**
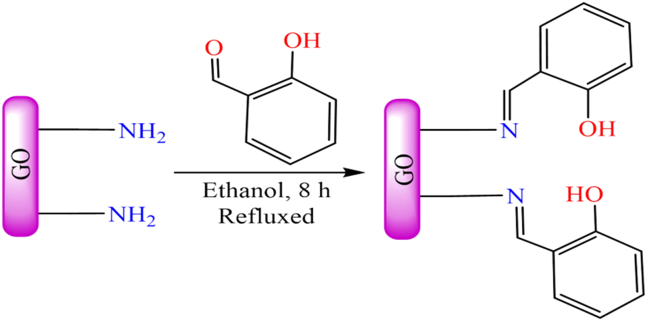
Synthetic procedure of graphene oxide supported salen legand Redrawn based on ref.^191^

**Figure 16:**
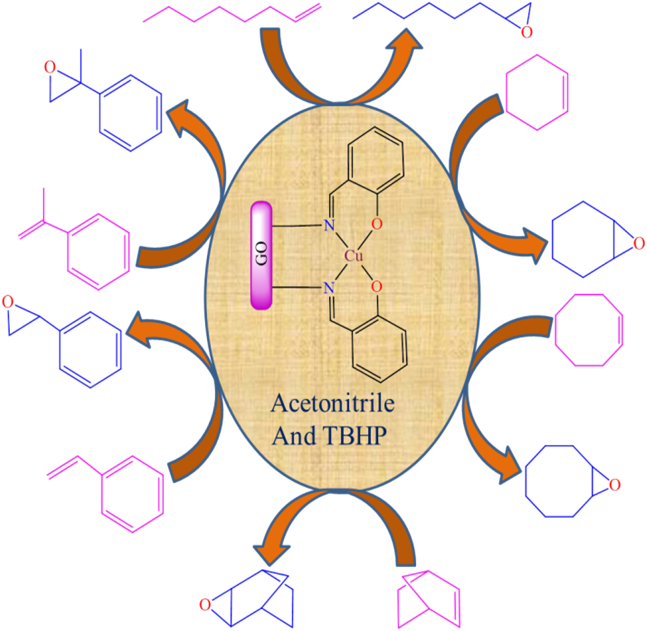
Figure show epoxidation of olefins catalyzed by copper-salen supported catalysts[Cu(salen)-f-GO]]

However, an efficient strategy was reported in 2014 to address the limitations of homogeneous catalysts by exploring *in situ* synthetic approaches for GO-immobilized metal salen complexes.^191^ The synthesis involved a condensation reaction between the amine groups of APTES-modified GO and the salen ligand, resulting in the covalently attached GO-immobilized salen ligand, as illustrated in Scheme 11. Although, immobilized ligands were further reacted with a copper salt, leading to the formation of a tetrahedral chelate, two-dimensional sheet-like Cu-complex ([Cu(salen)-f-GO]).^191^ The GO-supported copper complexes exhibited excellent catalytic efficiency in the epoxidation of various olefins, achieving yields of up to 98% under comparable reaction conditions (substrate: 10 mmol; catalyst: 60 mg; *tert*-BuOOH: 2.57 g; acetonitrile: 12 mL; temperature: 80°C; time: 12 h). As illustrated in Figure 16, the catalysts were tested for twelve consecutive cycles, with results indicating minimal loss in catalytic activity, demonstrating their stability and reusability in epoxidation reactions.

**Figure 17:**
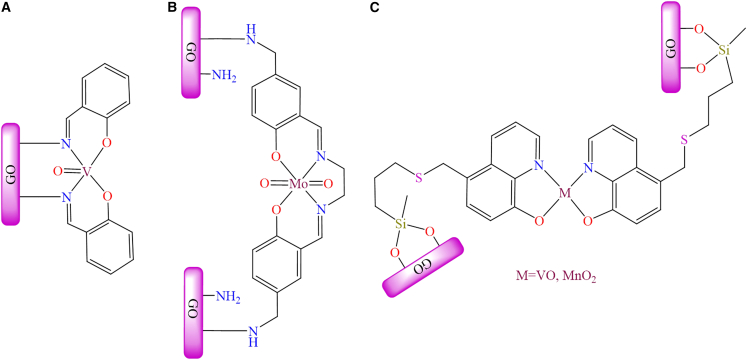
Figure represents supported molybdenum and vanadium complex (A and B) GO-Supported molybdenum complexes and (C) vanadium complex. Redrawn based on ref.^192^^,^^193^^,^^194^

Although, in 2012, H. P. Munges et al. synthesized a graphene-supported vanadium salen complex.^192^ Figure 17A illustrates the structure of the APTES-modified graphene oxide vanadium complex. This material served as a heterogeneous catalyst for the oxidation of various primary and secondary alcohols, *α*-hydroxyketones, and diols, affording the corresponding ketones and aldehydes in yields of up to 95% under the conditions: substrate (1 mmol), TBHP (1.5 mmol), catalyst (0.2 g), acetonitrile as solvent, at 65°C. Additionally, oxo-vanadium and dioxo-molybdenum Schiff base complexes were immobilized onto the surface of APTES-modified graphene oxide sheets and utilized as catalysts for the epoxidation of various alkenes using an oxidant.^193^^,^^194^ Figures 17B and 17C illustrates the GO-supported Mo and V complexes. These heterogeneous catalysts exhibited superior activity and reusability compared to their unsupported counterparts. Authors also described the catalytic mechanism of a metal complex for the epoxidation of cyclooctene in the presence of *t-*butyl hydroperoxide. The metal peroxo species was identified as the active component responsible for the catalytic epoxidation of olefins. The oxo-molybdenum supported catalyst achieved up to 93% conversion under the conditions: catalyst (50 mg; 5.0 mg for the neat catalyst), substrate (5 mmol), solvent (5 mL), oxidant (5 mmol), reaction time (8 h), and temperature (70°C). However, the oxo-vanadium supported catalyst afforded 96% conversion under optimized conditions: catalyst (10 mg), substrate (1 mmol), solvent (1 mL), oxidant (1 mmol), reaction time (8 h), and temperature (70°C).

## Merrifield resin as solid supports in heterogeneous catalysis

**Figure 18:**
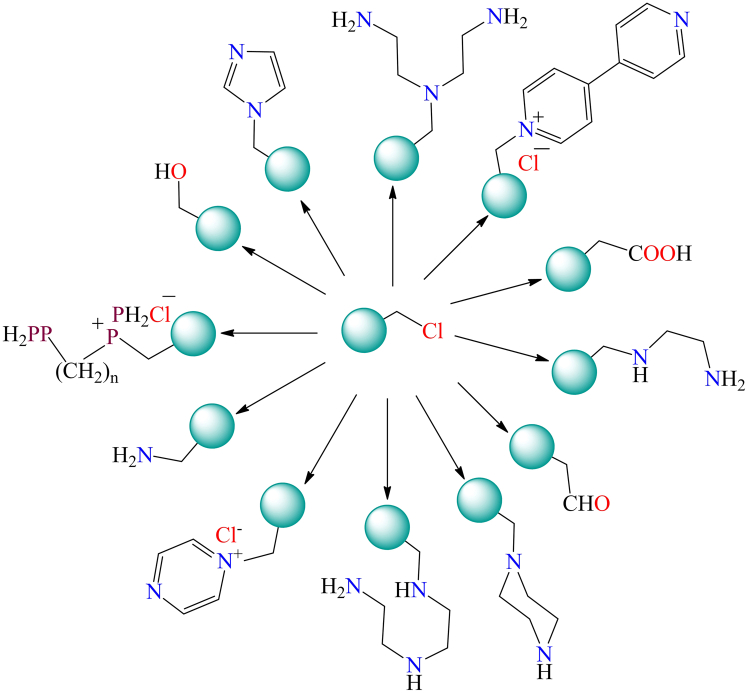
Figure illustrates the different functional modifications of chloromethylated polystyrene^135^

In 1984, Robert Bruce Merrifield got Noble prize for the invention of cross-linked polystyrene named Merrifield resin. They reports two types of functionalized Merrifield resin i.e., straight-chain and cross-linked polymer. The cross-linking nature of polystyrene resin influences its porosity, as well as its chemical and physical properties. Common cross-linking agents include divinyl sulfide, trichloroacetaldehyde (CCl_3_CHO), biphenols, disulfur dichloride (S_2_Cl_2_), diamines,^195^ ethylene glycol dimethacrylate (EGDMA),^196^ 1,4-bis(vinylphenoxy)butane,^197^ N,N-methylene bis(acrylamide) (MBA),^198^ trimethylpropane trimethacrylate (TRIM), and bis(vinylphenoxy) polyethylene glycol (PEG), among others.^199^ A polymer with more than 5% cross-linker is classified as macroporous, whereas one with less than 5% cross-linker is considered microporous, making it widely applicable in heterogeneous catalysis. Before use, Merrifield resin is typically swelled in an appropriate organic solvent, as swelling enhances interactions between the chloride (-Cl) groups in the resin and functional groups during the immobilization process. This allows for easy surface modification of the polymer resin with various reactive groups. Figure 18 illustrates the different functional modifications of chloromethylated polystyrene, showcasing its adaptability for various applications through the introduction of diverse reactive groups.^199^ Chloromethylated polystyrene that has undergone surface modification is widely utilized as a solid support for immobilizing homogeneous catalysts through covalent bonding. Polymer-grafted catalysts are gaining significant attention due to their non-volatility, non-toxicity, insolubility, and functional versatility, allowing them to function as heterogeneous catalysts while retaining the benefits of homogeneous catalysis.^200^^,^^201^ Compared to their solution-phase counterparts, polymer-supported metal catalysts offer several advantages, including ease of separation from the reaction mixture, recyclability, and reduced catalyst deactivation. These catalysts are particularly appealing for various organic transformation reactions.^3^^,^^202^ However, one major limitation of polymer-immobilized metal complexes is the issue of metal leaching. Despite this, the unique properties of polymer-grafted catalysts make them valuable for industrial applications.

**Figure 19:**
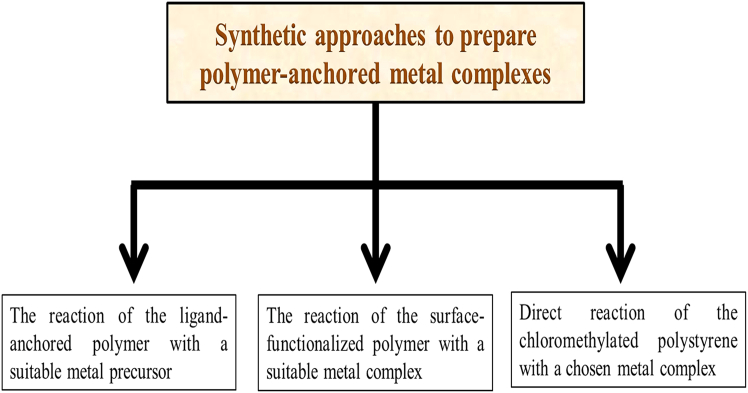
Types of synthetic procedure for the polymer anchored metal complex

In recent years, these catalysts have gained renewed interest for their potential in environmentally friendly green organic synthesis and their ability to deliver high activity and selectivity in catalytic processes.^203^^,^^204^ There are usually three techniques to synthesize polymer grafted metal complex (as shown in Figure 19), such as (1) the reaction of the ligand-anchored polymer with a suitable metal precursor, (2) the reaction of the surface-functionalized polymer with a suitable metal complex and, (3) direct reaction of the chloromethylated polystyrene with a chosen metal complex.

### The reaction of ligand-anchored polymer with a suitable metal precursor

The reaction of a ligand-anchored polymer with a suitable metal precursor is a widely used approach for synthesizing polymer-anchored metal complexes. This process typically involves multistep synthesis, where the first step is the preparation of polymer supported ligands and followed by the coordination of the metal precursor with the functional groups of the ligand immobilized polymer.^205^^,^^206^ The ligand-anchored polymer is reacted with the metal precursor in a suitable solvent (e.g., ethanol, methanol, or water) under controlled conditions (e.g., specific temperature and pH). Surface-functionalized polymer-metal complexes find applications in diverse fields, including organic synthesis, environmental remediation, and energy conversion processes.^207^ This approach aligns with the principles of green chemistry, as it enables the design of efficient, selective, and environmentally benign catalytic systems. Moreover, advancements in material science and polymer chemistry continue to expand the potential applications of these hybrid materials.^208^

**Scheme 12:**
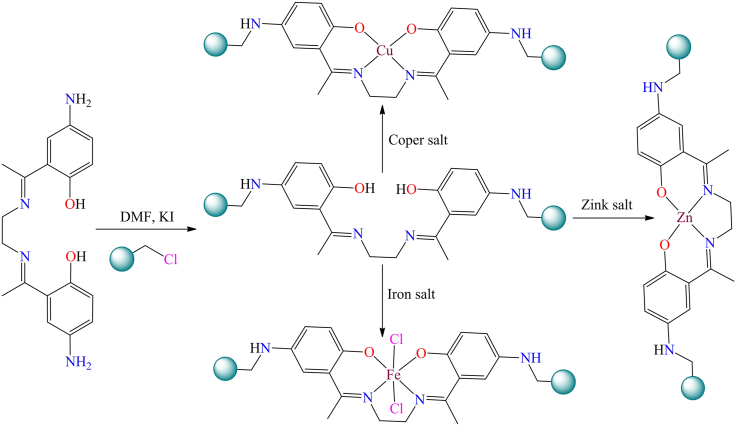
Scheme shows the step wise synthetic procedure of polymer supported metal complexes Redrawn based on ref.^209^

However, in 2008, K.C. Gupta et al. investigates the catalytic activity of polymer-supported and unsupported Schiff base complexes of Fe(III), Cu(II), and Zn(II) ions in the oxidation of phenol using hydrogen peroxide as an oxidant.^209^ Scheme 12 shows the stepwise synthetic procedure of polymer supported metal complexes. These polymer-supported copper, iron, and zinc complexes showed 49%, 70%, and 42% phenol conversion, respectively, under optimized reaction conditions: 0.05 mol phenol, 0.05 mol of 30 wt % aqueous hydrogen peroxide, at 70°C for 24 h. Among the three metal ions, Fe(III)-based complexes showed the highest catalytic activity, attributed to their ability to form highly reactive intermediate species. A proposed mechanism involves the formation of a peroxo complex (M-OOH) as the active species. The phenol interacts with this species to yield oxidation products through nucleophilic attacks at ortho and para positions.

**Figure 20:**
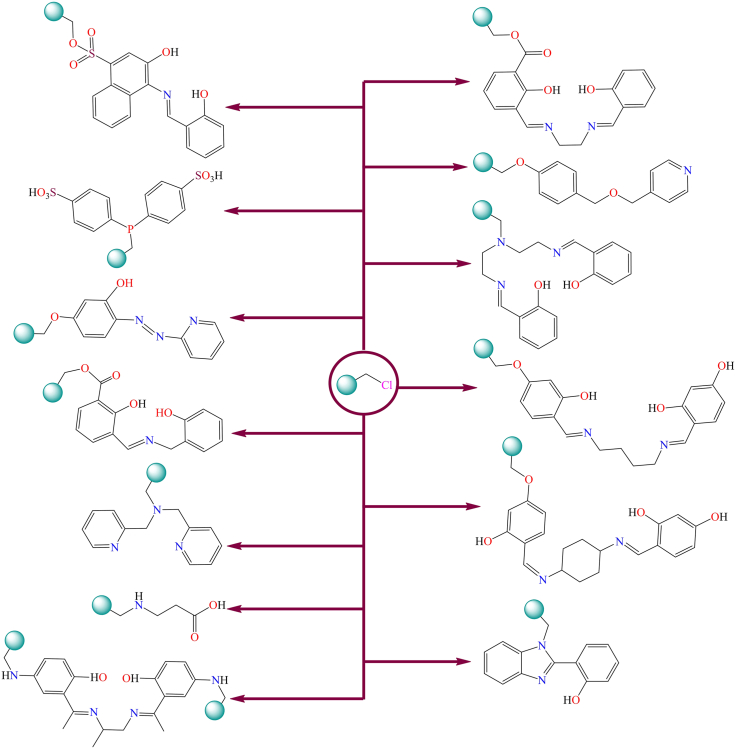
Anchoring of ligands on the surface of chloromethylated polystyrene by covalent linkage

Additionally, several synthetic methods have been documented in the literature for anchoring ligands onto the surface of chloromethylated polystyrene. A commonly employed approach involves the reaction of the -CH_2_Cl group in chloromethylated polystyrene with ligands or molecules containing reactive functional groups, such as carboxylic acid, sulfonic acid, amine, or hydroxyl groups.^210^^,^^211^ Mildly basic conditions are typically required for the reaction between the -CH_2_Cl group and molecules or ligands containing carboxylic or sulfonic acid groups. Similarly, hydroxyl groups react with chloromethylated polystyrene to form ether linkages under comparable reaction conditions.^6^^,^^212^^,^^213^^,^^214^^,^^215^^,^^216^^,^^217^^,^^218^ Some of the polymer supported ligands are shown in Figure 20.^6^^,^^212^^,^^213^^,^^214^^,^^215^^,^^216^^,^^217^^,^^218^ These chloromethylated polystyrene-supported ligands provides suitable sites for the coordination of various metals, including copper, nickel, cobalt, iron, zinc, cadmium, molybdenum, and uranium.

**Scheme 13:**
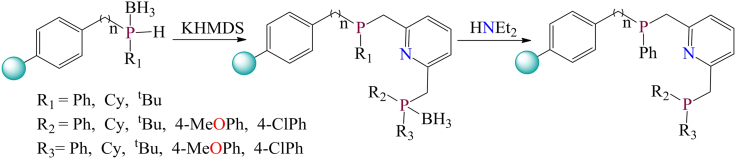
Scheme for the synthesis of phosphine based polymer anchored ligands Redrawn based on ref.^90^

**Figure 21:**
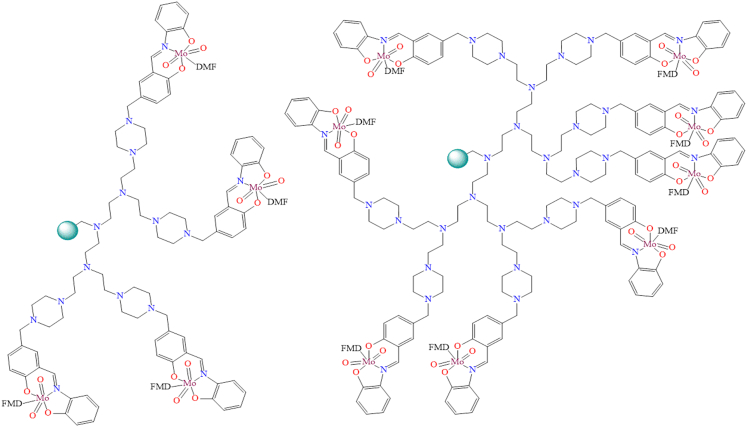
Figure illustrates the polymer-supported molybdenum complex Redrawn based on ref.^220^

However, for the synthesis of phosphine-based polymer-supported ligands, chloromethylated polystyrene is directly reacted with suitable phosphine ligands.^3^^,^^219^ Recently, several polymer-immobilized non-symmetrical PNP pincer ligand systems were reported in the literature by Robert Konrath et al. and tested for catalytic ester hydrogenation (Scheme 13).^90^ Additionally, Weili Wang et al. developed polymer resin-grafted Schiff base molybdenum complexes as efficient catalysts for the three-component, one-pot synthesis of pyrrole-*2*-one derivatives at room temperature, yielding the desired products.^220^ Figure 21 illustrates the polymer-supported molybdenum complex. The supported catalysts were synthesized through a stepwise approach. The authors concluded that aromatic aldehydes and amines with electron-donating groups lead to shorter reaction times and higher yields compared to those with electron-withdrawing groups. This method offers a simple and effective approach for rapidly synthesizing a highly structured and diverse molecular library. The use of these catalysts substantially increased product yields (up to 95%) and shortened reaction times from 6 h to 2 h under comparable conditions, employing amine (2 mmol), aldehyde (1 mmol), ethyl pyruvate (3 mmol), and catalyst (0.5 mol %). Additionally, the catalysts retain their catalytic activity even after being recycled and reused for up to eight cycles.

### The reaction of surface-functionalized polymer with a suitable metal complex

**Figure 22:**
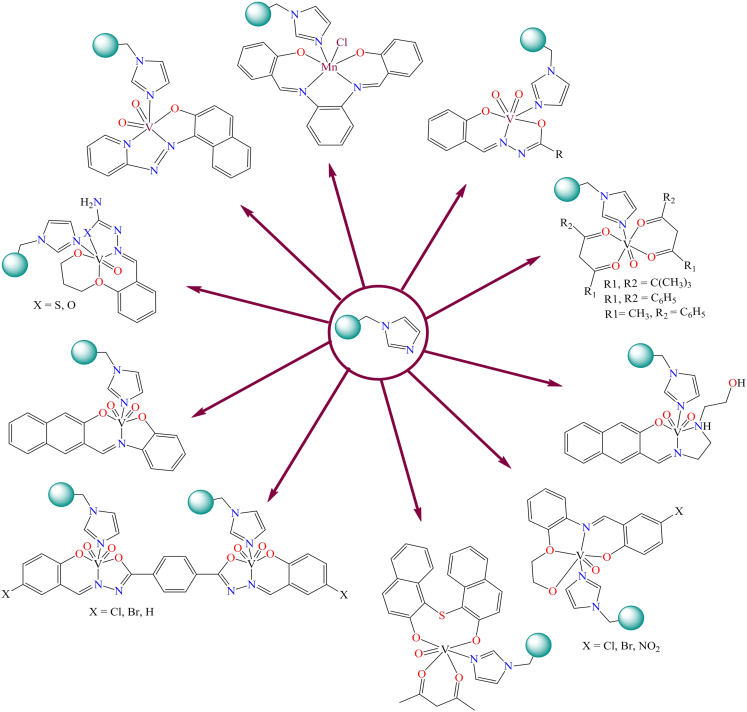
Structures of various imidazole modified polymer resin grafted metal complexes

The functionalization of polymer surfaces and their subsequent reaction with metal complexes have emerged as significant strategies in the development of advanced materials for catalysis and other industrial applications. The surface-functionalized polymers provide a versatile platform for anchoring metal complexes through covalent bonding, which enhances the stability, reusability, and activity of the resulting materials. This approach allows for the fine-tuning of catalytic properties by modifying the functional groups on the polymer surface and selecting appropriate metal precursors, offering a sustainable alternative to conventional homogeneous catalysts. The process involves the introduction of functional groups, such as amines, carboxyls, or phosphines, onto the polymer backbone, which act as coordination sites for the metal complexes (as shown in Figure 18). These functional groups facilitate the immobilization of metal centers, leading to improved recyclability and reduced metal leaching in catalytic systems.^221^^,^^222^ The formation of polymer-grafted metal complexes via the reaction between surface-functionalized polymers and metal complexes is relatively uncommon and depends on specific conditions. Among the various surface modifications, amine-functionalized polymers are the most frequently used for this approach. The nitrogen in imidazole can directly coordinate with metals, making imidazole-modified Merrifield resin a significant carbonaceous support in heterogeneous catalysis. Figure 22 illustrates different types of metal complexes immobilized on imidazole-modified Merrifield resin through covalent bonding.^223^^,^^224^^,^^225^^,^^226^^,^^227^^,^^228^^,^^229^^,^^230^^,^^231^^,^^232^ The authors used imidazole-modified Merrifield-supported complexes as heterogeneous catalysts for organic transformation reactions.

**Scheme 14:**
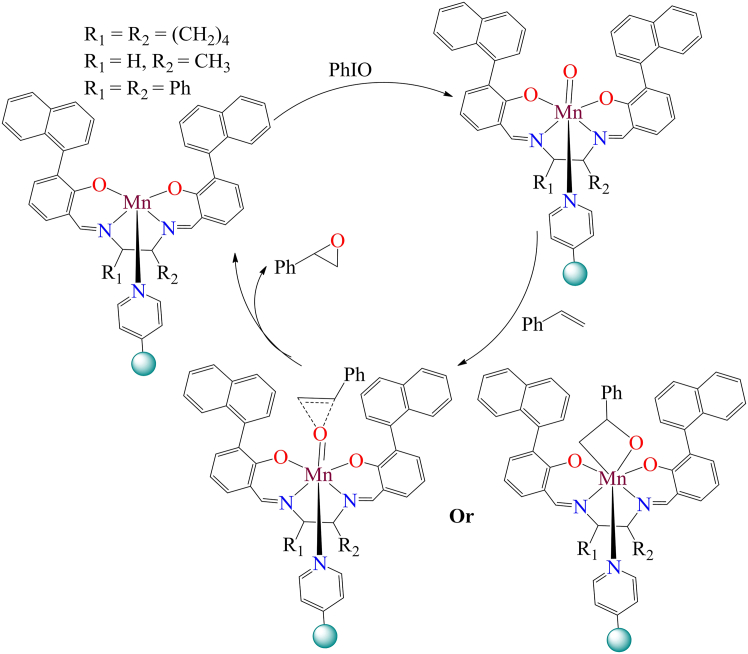
The reaction mechanism involves the formation of a Mn-oxo complex for styrene epoxidation

Although, in 1997, R.I. Kureshy et al. introduced chiral Mn complexes grafted onto pyridine-modified polymer resin.^233^ These complexes were synthesized using *(1R,2R)-(−)-*diphenylethylenediamine, *(1S,2S)-(+)-*cyclohexanediamine, and *(S)-(+)-1*-amino propane, in combination with *α*-naphthyl salicylaldehyde, before being anchored onto a modified polymeric matrix. The resulting catalysts were employed for the enantioselective epoxidation of styrene and substituted styrenes, achieving 25%–65% conversion using 0.02 mmol catalyst, 1 mmol styrene derivatives, 0.1 mmol n-tridecane, 5 mL dichloromethane, and 1 mmol iodosylbenzene, with a reaction time of 30 min. They proposed reaction mechanism involves the formation of a Mn-oxo complex, which facilitates oxygen transfer from a reactive Mn-oxo intermediate to styrene (illustrated in Scheme 14). Notably, these catalysts demonstrated recyclability, maintaining their activity for at least ten cycles.

Additionally, in 2015, Mannar R. Maurya et al. published a review in Coordination Chemistry Reviews that examined various methods for immobilizing vanadium complexes on different solid supports.^234^ Their work primarily focused on strategies employing mesoporous materials, such as polymers and zeolites, and explored the catalytic applications of these systems. The study detailed the use of polymer-supported complexes in organic transformation reactions and described how supported vanadium catalysts are typically synthesized through the covalent attachment of the metal complex to the support via several anchoring techniques. Furthermore, the review noted that the active metal species within these heterogeneous catalysts can be established through mechanisms, such as adsorption, covalent bonding between the metal ligand and the support, ion exchange, encapsulation, or entrapment. Recently, Harish Kumar Chopra et al. published a review on Merrifield resin-supported metal complexes and their applications in organic transformations.^235^

### Direct reaction of chloromethylated polystyrene with a chosen metal complex

**Figure 23:**
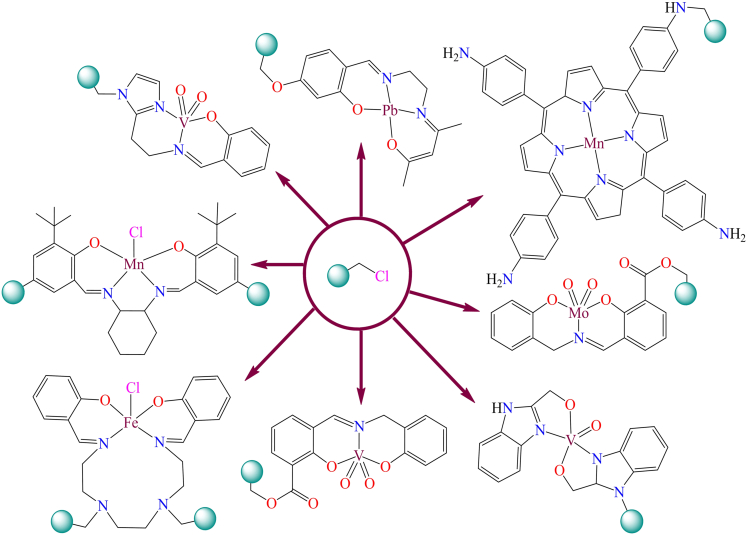
Examples of polymer anchored transition metal complexes through direct methods

The third method offers a single-step process, the first two synthetic approaches are more frequently utilized for preparing the majority of polymer-supported metal complexes. Directly reacting metal complexes with Merrifield resin is a simple and straightforward procedure, but it is the least commonly used approach. For this method, the metal complex must possess a reactive functional group or atom that can interact with the -CH_2_Cl groups of chloromethylated polystyrene. Furthermore, this technique is unsuitable for complexes that are thermally unstable. Listed below are examples of polymer-grafted catalysts prepared through the direct reaction of metal complexes with Merrifield resin which used for the organic transformation reaction (Figure 23).^236^^,^^237^^,^^238^^,^^239^^,^^240^

**Figure 24:**
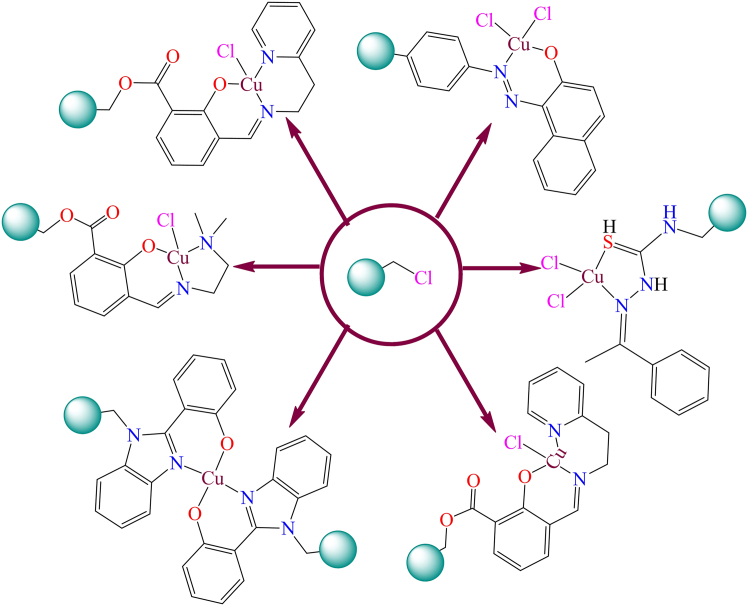
Polymer supported copper complexes used for the heterogeneous catalytic oxidation of alkenes

Although, Copper-based heterogeneous catalytic oxidation of alkenes is a well-explored area in organic chemistry and industrial applications. Chloromethylated polystyrene-anchored catalysts play a significant role in the selective oxidation of alkenes, producing valuable compounds such as epoxides, aldehydes, ketones, and carboxylic acids with the help of solid catalysts and suitable oxidizing agents. These reactions are vital for synthesizing fine chemicals, pharmaceuticals, and polymer precursors. Some examples of mononuclear copper complexes used in heterogeneous catalytic oxidation of alkenes are illustrated in Figure 24.^91^^,^^241^^,^^242^^,^^243^

**Figure 25:**
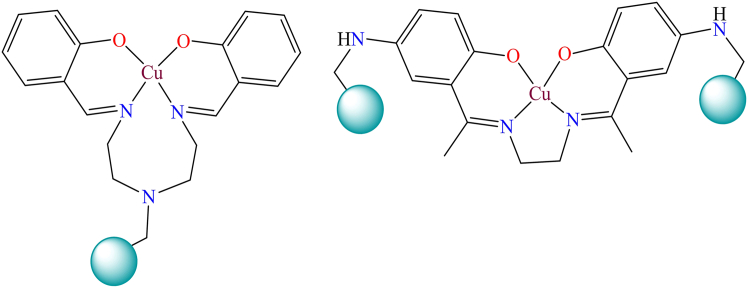
Figure shows polymer-supported copper complexes^209^^,^^244^ Copyright © 2008, Elsevier.

However, K. C. Gupta et al. reported various copper complexes that were immobilized on Merrifield resin.^209^^,^^244^ During the immobilization process, chloromethylated polystyrene polymer was directly reacted with copper complexes. Figure 25 shows several polymer-supported copper complexes. They used the supported complexes as heterogeneous catalysts for the oxidation of alcohols in the presence of oxidants. They also studied the effect of hydrogen peroxide on the copper complexes and found that copper peroxo species were active for the oxidation of alcohols.

**Figure 26:**
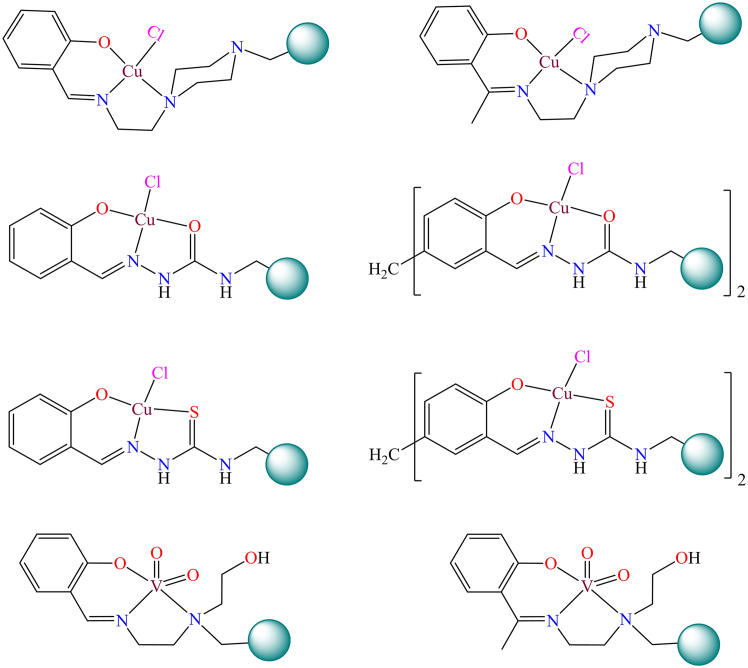
Figure shown various polymer immobilized copper and vanadium complexes for oxidation of various organic compounds Redrawn based on ref.^245^^,^^246^

Furthermore, C. Haldar et al. synthesized various polymer-anchored vanadium and copper complexes for oxidation and bromination reactions of organic compounds. They employed a direct approach, wherein Merrifield resin was directly reacted with metal complexes. Figure 26 illustrates the polymer beads anchored with vanadium and copper.^17^^,^^245^ These supported metal complexes function as heterogeneous catalysts for the oxidative bromination of organic compounds and the oxidation of thioethers under specific reactin conditions. However they anchored some mono and binuclear copper compexes on Merrifield resin through direct techniques for oxidation of various alkenes. Figure 26 shown various polymer immobilized copper complexes.^246^ The supported copper complexes used for liquid phase oxidation or epoxidation of various alkenes in presence of peroxide under optimized condition. The anchored catalysts have high activity and selectivity as compared to neat catalysts, is due to high surface area which provide path for the interaction between substrate and catalysts.

Additionally, Sk. M. Islam et al. developed a novel polymer-supported Cu(II) complex, which was evaluated for its effectiveness in sulfide oxidation and oxidative bromination reactions using hydrogen peroxide as the oxidant.^247^ The study demonstrated the oxidation of various sulfides and the oxidative bromination of organic substrates. Authors used this catalyst for the oxidation of sulfides and their derivatives, reporting activity with 68%–91% substrate conversion under the following reaction conditions: 5 mmol substrate, 10 mmol of 30% H_2_O_2_, and 10 mL CH_3_CN at room temperature. In another study, the catalyst was applied for oxidative bromination of various substrates, achieving 12%–97% conversion under specific conditions: 2 mmol substrate, 2.2 mmol KBr, 5 mL glacial acetic acid, and 5 mmol of 30% aqueous H_2_O_2_ at room temperature. These polymer-supported Cu(II) catalyst could be easily recovered through simple filtration and reused more than six times without significant loss of its initial activity. Additionally, In 2018, B. Altava et al. published a review article discussing chiral catalysts immobilized on polymers to enhance their efficiency.^6^ Their review examined numerous polymer-supported chiral catalytic systems from existing research. The examples provided clearly demonstrate that the support material significantly influences the performance of these systems rather than being a passive component. Therefore, considering the support as a crucial factor in designing chiral immobilized catalysts is essential. By carefully selecting and optimizing the physicochemical properties of the polymeric support, it is possible to improve key attributes of the catalytic system, such as activity, stability, and selectivity, including enantioselectivity in chiral systems. However, further research is needed to gain deeper insight into the molecular mechanisms underlying these beneficial support effects. Such understanding will enable the rational design of more advanced and efficient polymer-supported chiral catalysts in the future. However, in 2022, Tungabidya Maharana et al. published a review article in Reactive and Functional Polymers, summarizing recent advancements in the synthesis and catalytic applications of both unsupported and polymer-supported first-row transition metal Schiff base complexes for alkene epoxidation.^248^ The review highlights the use of polymer-supported Schiff base complexes of V, Mn, Fe, Co, Ni, Cu, and Zn as catalysts for the epoxidation of various alkenes, including limonene, cyclohexene, styrene, *cis*-stilbene, *trans*-stilbene, verbenone, linear alkenes, cyclooctene, α-methyl styrene, and α-pinene. The authors also explores the catalytic performance of these polymer-supported metal complexes, taking into account the diverse Schiff base ligands and oxidants used in epoxidation reactions. The supported catalysts demonstrated significantly higher conversion efficiencies, ranging from approximately 80%–95%, whereas the corresponding unsupported complexes achieved comparatively lower conversions, in the range of about 50%–75%. This enhanced catalytic performance of the supported systems may be attributed to better dispersion of the active sites, increased surface area, and improved stability of the catalytic complexes upon immobilization. Additionally, the recyclability of both polymer-supported and unsupported Schiff base complexes of 3d transition metals is examined to provide further insights into their stability and efficiency. Based on existing literature, the authors present a mechanistic overview of the epoxidation process. This review serves as a valuable resource for researchers in materials science and catalysis, offering a comparative analysis of polymer-supported versus unsupported catalysts and their unique advantages.

## Carbon nitride as a solid support in heterogeneous catalysis

**Scheme 15:**
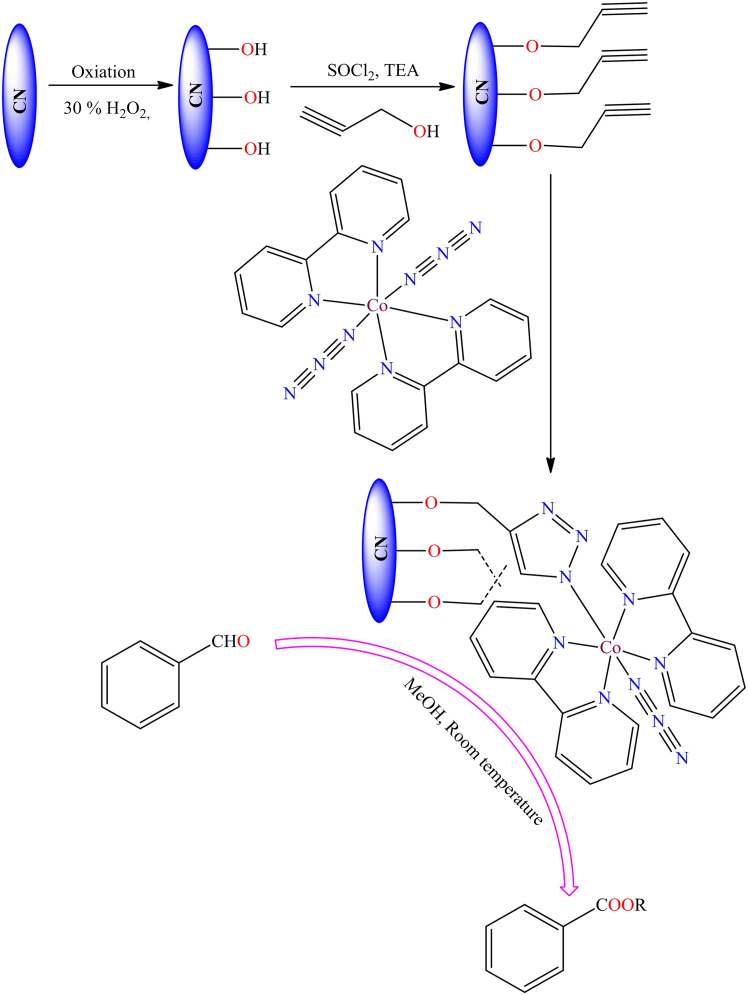
A nanoporous graphitic carbon nitride supported cobalt bipyridine complex act as heterogenized photocatalyst for the direct esterification of aldehydes under visible light at room temperature (benzaldehyde (1.0 mmol), methanol (5 mL), time 12 h) Redrawn based on ref.^253^

Graphitic carbon nitride (g-C_3_N_4_) is a polymeric material composed of triazine (C_3_N_3_) or heptazine (C_6_N_7_) units interconnected through covalent carbon-nitrogen bonds. This arrangement forms a two-dimensional framework similar to graphene, but with nitrogen incorporation. Due to its exceptional physicochemical properties, high thermal and chemical stability, and adjustable electronic structure, carbon nitride (C_3_N_4_) has gained recognition as a promising solid support material. It is widely applied in catalysis, adsorption, and energy storage.^249^ Its extended conjugated network and nitrogen-rich composition enhance its ability to coordinate with metal species, making it highly suitable for metal-supported catalysis, photocatalysis, and heterogeneous catalytic reactions.^250^ Given its remarkable stability and adaptable electronic properties, carbon nitride serves as an effective support in diverse catalytic and adsorption-based applications.^251^^,^^252^ Carbon nitride has been extensively utilized as a support material for transition metal catalysts in various organic synthesis reactions. However, A cobalt bipyridine complex containing an azide group was covalently anchored onto propargyl-functionalized nanoporous graphitic carbon nitride (npg-C_3_N_4_) using a click reaction (shown in Scheme 15).^253^ This process yielded a heterogenized photocatalyst that efficiently promoted the direct esterification of aldehydes, achieving a 95% product yield under visible light irradiation at room temperature in 5 mL of methanol within 12 h. Employing the click reaction as a grafting approach ensured strong covalent attachment of the cobalt complex to the support, leading to higher catalyst loading while preventing leaching. Additionally, the carbon nitride support contributed to a synergistic effect, enhancing the reaction rate. The mildly basic nature of the nitrogen-rich graphitic structure enabled ester synthesis without requiring an external base. The resulting photocatalyst demonstrated excellent stability, allowing for easy recovery and multiple reuse cycles without significant loss of activity.

**Figure 27:**
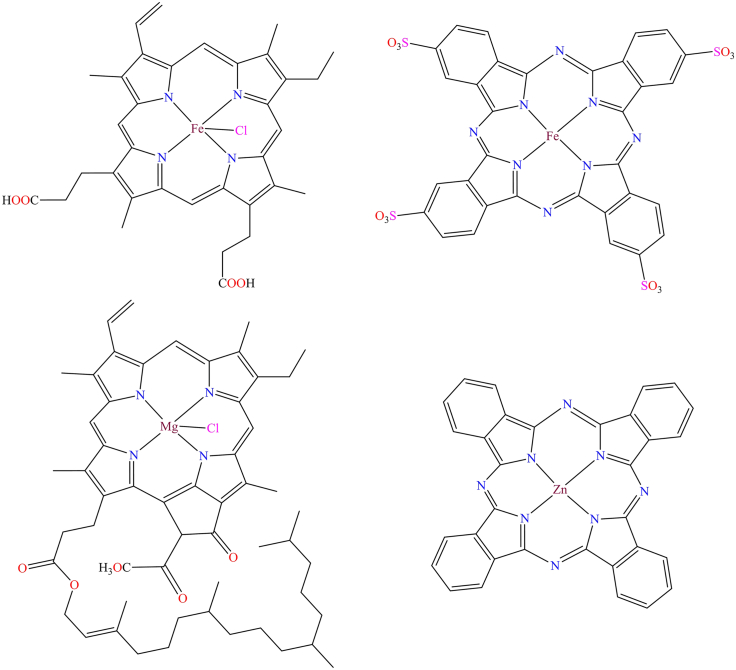
Porphyrin based metal complexes which produced organic-metal hybrid material with g-C_3_N_4_ Redrawn based on ref.^254^

**Figure 28:**
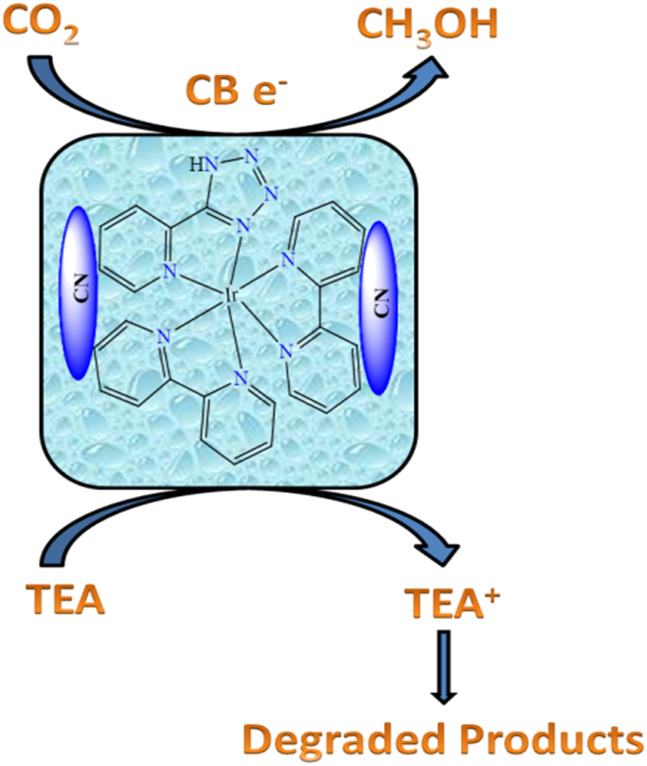
Possible mechanistic pathway for photoreduction of CO_2_ using npg-C_3_N_4_ based Ir complex

Moreover, in 2009, X. Wang et al. introduced a novel functional organic-metal hybrid material in Advanced Materials.^254^ They synthesized porphyrin-based metal complexes and immobilized them on graphitic carbon nitride, as depicted in Figure 27. The materials underwent comprehensive characterization using X-ray diffraction (XRD), X-ray photoelectron spectroscopy (XPS), optical absorption spectroscopy, thermal analysis, elemental analysis, and Fourier transform infrared (FT-IR) spectroscopy. The study suggested that incorporating various metals with optical, electronic, magnetic, and catalytic properties into the g-C_3_N_4_ framework could lead to the development of innovative functional organic-metal hybrid materials for advanced technological applications. The Figure 28 illustrates an efficient and selective system for the photocatalytic reduction of CO_2_ to methanol under visible light irradiation.^255^ This system was developed using a metal-free semiconductor, npg-C_3_N_4_, in combination with an iridium complex. After 24 h of irradiation, the catalyst demonstrated a high turnover number (TON) of 1241 for iridium when triethylamine (TEA) was used as a sacrificial electron donor. The presence of TEA was found to be crucial for achieving a higher methanol yield selectively, with no detectable formation of other gaseous or liquid byproducts. Furthermore, the photocatalyst maintained nearly consistent activity over six consecutive cycles, yielding methanol with similar efficiency in each run. This finding highlights the potential of immobilizing metal complexes onto photoactive semiconductor supports, paving the way for the development of highly efficient photocatalysts for the sustainable conversion of CO_2_ into valuable products.

**Figure 29:**
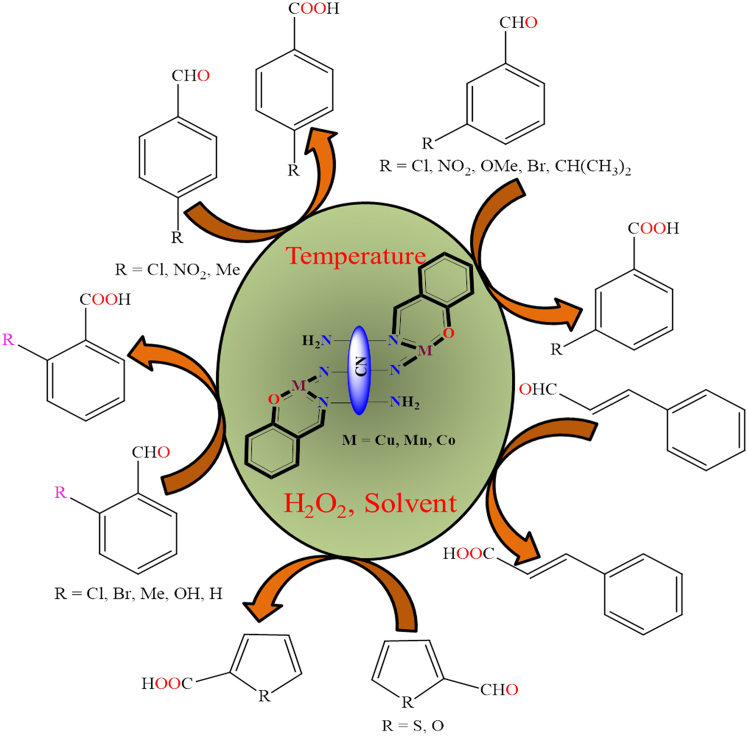
Oxidation of aldehydes and its derivative in presence of supported catalysts using H_2_O_2_ under mild reaction conditions and short reaction times

Furthermore, to develop environmentally friendly catalysts for organic transformations, a novel series of reusable M(salen)@g-C_3_N_4_ catalysts (M = Co, Cu, Mn) was synthesized by incorporating metal-salen complexes onto a g-C_3_N_4_ support.^256^ These catalysts exhibited remarkable efficiency in oxidizing aldehyde derivatives using H_2_O_2_ under mild reaction conditions and short reaction times (as shown in Figure 29). Among them, the Co(salen)@g-C_3_N_4_ catalyst demonstrated the highest catalytic activity and was further optimized for oxidation reactions. The catalysts showcased excellent efficiency, durability, and recyclability, making them well-suited for extended use. Additionally, the method proved to be robust and effective for synthesizing benzoic acid derivatives on a larger scale. Its scalability was confirmed by achieving high isolated yields on a gram scale, highlighting its reliability for producing significant quantities of benzoic acid derivatives.

**Scheme 16:**
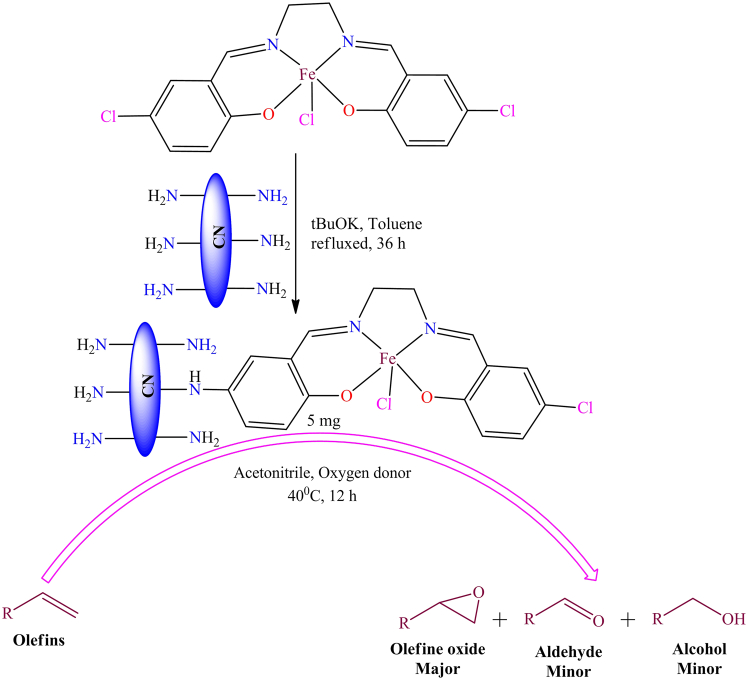
Synthesis of hybrid catalyst [C_3_N_4_-FeCl(Salen)]_Chem_ and used as epoxidation catalysts for various olefins Redrawn based on ref.^257^

Moreover, Sebastiano Gadolini et al. developed a novel catalytic system, [C_3_N_4_-FeCl(Salen)]_Chem_, for the photocatalytic epoxidation of various olefins.^257^ Scheme 16 shows the hybrid catalyst, which was created by anchoring iron salen-type complexes onto graphitic carbon nitride (C_3_N_4_), resulting in enhanced catalytic efficiency, particularly in terms of epoxide selectivity and recyclability. It exhibited epoxidation activity with both cyclic and linear olefins, including styrene, cyclohexene, *α*-pinene, *1*-octene, and *cis*-*4*-octene. The approach utilized the intrinsic amino-terminal groups within C_3_N_4_ as anchoring sites, eliminating the need for additional linkers or heterojunction formation with other semiconductors. While the catalyst demonstrates promising performance and recyclability, it may not be the most active or stable option available.

However, recently, Ivo F. Teixeira et al. published a review article in Chemical Society Reviews discussing single-atom catalysis (SAC) using carbon nitride-supported materials.^258^ Their work highlights how SAC bridges the gap between homogeneous and heterogeneous catalysis. However, the field faces significant challenges, particularly in controlling the bonding and coordination between single atoms and the support material. This is crucial for stabilizing single atoms and compensating for the increased surface energy resulting from atomic dispersion. However, the interaction between single atoms and carbon nitride supports is bidirectional single atoms can modify the electronic properties of the carbon nitride matrix, the matrix itself can influence the catalytic activity of the single sites in photocatalytic reactions. Carbon nitride materials, with their nitrogen-rich coordination sites, effectively trap metal atoms, making them promising hosts for single-atom catalysts (SACs). The review explores recent advancements in SACs supported by carbon nitride, covering key characterization techniques, challenges in the field, and commonly used synthetic methods for different carbon nitride materials. Additionally, it examines the catalytic performance of SACs, particularly in photocatalytic applications. The authors also discuss emerging frontiers in the field, including the development of advanced analytical techniques, precise synthetic methods for fine-tuning metal loading and multi-element synthesis, and a deeper understanding of the reciprocal interaction between single atoms and carbon nitride supports, which could drive future innovations.

## Research gap, future direction, and challenges

The surveyed studies highlight several critical gaps in current research on immobilized Schiff base metal complexes supported on carbon materials. While numerous reports demonstrate improvements in catalytic activity, selectivity, and recyclability, there is a noticeable absence of quantitative relationships linking structural factors, such as metal dispersion, loading, and surface functionalities, to catalytic metrics like TON, turnover frequency TOF, and selectivity.^259^^,^^260^ In addition, the mechanistic understanding remains insufficient, largely due to the limited application of advanced *in situ* and *operando* analytical tools, including X-ray absorption spectroscopy (XAS), X-ray photoelectron spectroscopy (XPS), and diffuse reflectance infrared Fourier transform spectroscopy (DRIFTS). Consequently, the influence of immobilization on the active sites’ electronic environment, oxidation state, and accessibility is still not fully understood.^261^^,^^262^ Furthermore, inconsistencies in catalyst leaching, hot-filtration, and recyclability evaluations, along with the scarcity of long-term performance tests under industrially realistic conditions, hinder a clear assessment of catalyst durability and the separation of heterogeneous versus homogeneous catalytic effects.^263^^,^^264^ Despite significant progress in immobilizing Schiff base metal complexes on carbon-based supports, several critical challenges and knowledge gaps remain. Although many studies report enhanced catalytic activity and recyclability, few establish robust quantitative relationships between catalytic performance and structural parameters such as metal loading, particle dispersion, and surface functionality.^265^^,^^266^ The mechanistic understanding of how immobilization influences the electronic structure, active-site environment, and reaction pathway is still limited, largely due to the insufficient application of *in situ* and *operando* spectroscopic techniques.^267^ A key debate persists over whether catalytic activity originates from the immobilized heterogeneous sites or from soluble species leached into the reaction medium, as tests such as leaching analysis, hot filtration, and poisoning experiments are not consistently performed. Additionally, the underlying cause of improved performance whether arising from metal-support electronic interactions or from confinement and stabilization effects remains unresolved.^259^^,^^260^ Another notable research gap involves the limited evaluation of these catalysts under industrially relevant conditions, including long-term durability, mass-transfer limitations, and regeneration in continuous processes. Integrating Schiff base coordination chemistry with single-atom catalyst and metal-organic hybrid frameworks could yield materials that combine molecular-level tunability with structural robustness.^263^^,^^264^ Addressing these issues through coordinated, systematic, and standardized studies will be essential for translating Schiff base-based catalysts into practical industrial applications. Moreover, developing unified characterization and benchmarking protocols with consistent reporting of parameters, such as metal loading, turnover number, recyclability, and leaching data will enable meaningful cross-study comparisons and help distinguish genuinely heterogeneous systems from those influenced by homogeneous leaching.^259^^,^^260^

An emerging direction in this field focuses on integrating Schiff-base coordination chemistry with single-atom and dual-site catalyst designs supported on materials such as g-C_3_N_4_, graphene oxide, and nitrogen-doped carbons. Atomically dispersed metal centers maximize metal utilization while allowing precise tuning of their coordination environments. The introduction of nitrogen vacancies or tailored dopant distributions can further stabilize active sites and modulate their electronic properties, thereby improving catalytic selectivity.^263^ Coupling advanced experimental techniques with computational approaches particularly density functional theory (DFT) and machine learning (ML) provides a powerful framework for predicting optimal support-ligand-metal architectures and identifying key descriptors governing activity, selectivity, and stability. From an application standpoint, moving from conventional batch reactions to continuous-flow systems under realistic industrial conditions is a critical step forward. Comprehensive assessments of catalyst lifetime, regeneration behavior, and mass-transfer limitations will yield valuable insights into scalability. In parallel, evaluating the environmental and economic viability of these systems through life-cycle assessment (LCA) and techno-economic analysis (TEA) will inform sustainable catalyst design. Another major challenge is the chemical instability of imine linkages, which are prone to hydrolysis and oxidation. Developing more robust immobilization strategies such as polymer-supported or framework-embedded Schiff-base architectures, can significantly enhance catalyst durability and reduce metal leaching.^268^^,^^269^ Altogether, these integrated approaches will strengthen mechanistic understanding, improve structural resilience, and accelerate the advancement of Schiff-base-derived catalysts toward sustainable industrial implementation.

## Conclusion

This review highlights recent advances in the development of supported metal-Schiff base catalysts immobilized on carbonaceous polymeric matrices, offering a promising route to bridge homogeneous and heterogeneous catalysis. By anchoring transition metal complexes onto supports like activated carbon, carbon nanotubes, graphene, Merrifield resin, and graphitic carbon nitride, researchers have achieved enhanced catalytic activity, stability, and reusability across various organic transformations. The immobilization of metal catalysts onto carbonaceous polymeric supports has emerged as a powerful strategy for enhancing catalytic performance. The review emphasizes the advantages of carbonaceous supports, such as tunable surface properties, high surface area, and chemical versatility, and addresses challenges like metal leaching and activity shifts post-immobilization. Metal leaching poses a major challenge in catalytic processes, especially in heterogeneous systems and supported metal-based catalysts. To address this issue, several approaches can be employed, including enhancing metal-support interactions, designing core-shell architectures, utilizing chelating ligands, and fine-tuning reaction conditions to improve catalyst immobilization. This review explains various immobilization techniques used to strengthen the interaction between metal complexes and solid supports, thereby reducing metal leaching during catalysis. It also outlines and future directions including green synthesis, and the strategic design of support materials, positioning polymer-supported catalysts as key players in advancing sustainable and industrially viable catalytic systems. Overall, Schiff-base metal complexes on carbon-based supports show great catalytic potential, but gaps remain in mechanistic understanding, stability, and industrial applicability. Future work should focus on *in situ*/operando studies, standardized benchmarking, and advanced designs like single-atom or dual-site catalysts to enhance activity and durability. Integrating computational modeling and continuous-flow testing will further guide optimization. Addressing these challenges is key to translating laboratory success into sustainable industrial applications.

## Acknowledgments

The authors express their sincere gratitude to all researchers and scientists whose work has contributed to the advancements in supported metal catalysis. We are grateful to all the scientists and researchers who contributed to this article, either directly or indirectly. Authors are grateful to Department of Chemistry, Galgotias University for their logistical or administrative support. We are grateful to all non-author contributors and to the funding sources whose data we have used for the completion of this manuscript. Additionally, we acknowledge the contributions of laboratories and non-author such as technical staff for their assistance in data analysis. Lastly, we appreciate the support of our families and friends, whose encouragement has been instrumental in the completion of this review. The author also acknowledges Ms. Priyanka Maurya and Ms. Dimpal Maurya for drawing some of the images. The authors would also like to thank all the interviewees and survey participants for their input and cooperation.

## Author contributions

Conceptualization, methodology, writing-original draft, writing-review & editing: A.M.

## Declaration of interests

There are no conflicts of interest to declare.
